# Interplay of myosin phosphatase and protein phosphatase-2A in the regulation of endothelial nitric-oxide synthase phosphorylation and nitric oxide production

**DOI:** 10.1038/srep44698

**Published:** 2017-03-16

**Authors:** Róbert Bátori, Bálint Bécsi, Dénes Nagy, Zoltán Kónya, Csaba Hegedűs, Zsuzsanna Bordán, Alexander Verin, Beáta Lontay, Ferenc Erdődi

**Affiliations:** 1 Department of Medical Chemistry, Faculty of Medicine, University of Debrecen, Debrecen, Egyetem tér 1, H-4032, Hungary; 2 MTA-DE Cell Biology and Signaling Research Group, Faculty of Medicine, University of Debrecen, Debrecen, Egyetem tér 1, H-4032, Hungary; 3 Vascular Biology Center, Division of Pulmonary Medicine, Medical College of Georgia, Augusta University, Augusta, GA, USA

## Abstract

The inhibitory phosphorylation of endothelial nitric oxide (NO) synthase (eNOS) at Thr497 (eNOS^pThr497^) by protein kinase C or RhoA-activated kinase is a major regulatory determinant of eNOS activity. The signalling mechanisms involved in the dephosphorylation of eNOS^pThr497^ have not yet been clarified. This study identifies myosin phosphatase (MP) holoenzyme consisting of protein phosphatase-1 catalytic subunit (PP1c) and MP target subunit-1 (MYPT1) as an eNOS^pThr497^ phosphatase. In support of this finding are: (i) eNOS and MYPT1 interacts in various endothelial cells (ECs) and in *in vitro* binding assays (ii) MYPT1 targets and stimulates PP1c toward eNOS^pThr497^ substrate (iii) phosphorylation of MYPT1 at Thr696 (MYPT1^pThr696^) controls the activity of MP on eNOS^pThr497^. Phosphatase inhibition suppresses both NO production and transendothelial resistance (TER) of ECs. In contrast, epigallocatechin-3-gallate (EGCG) signals ECs via the 67 kDa laminin-receptor (67LR) resulting in protein kinase A dependent activation of protein phosphatase-2A (PP2A). PP2A dephosphorylates MYPT1^pThr696^ and thereby stimulates MP activity inducing dephosphorylation of eNOS^pThr497^ and the 20 kDa myosin II light chains. Thus an interplay of MP and PP2A is involved in the physiological regulation of EC functions implying that an EGCG dependent activation of these phosphatases leads to enhanced NO production and EC barrier improvement.

Endothelium-derived nitric oxide (NO) is an important gaseous mediator of numerous cardiovascular functions. The synthesis of NO from L-arginine is catalyzed by endothelial NO synthase (eNOS), therefore the regulation of eNOS activity is a key factor in the physiological control of NO level in the cells^1^. The activity of eNOS is dependent on its subcellular localization^2^,^3^ and is also mediated by protein-protein interactions as well as by posttranslational modifications^4^,^5^.

Among the posttranslational processes phosphorylation of eNOS at serine (Ser) and threonine (Thr) residues bears special importance in the regulation of enzyme activity^6^. The major phosphorylation sites identified in eNOS are Ser116, Thr497, Ser617, Ser635 and Ser1179 (numbering the residues are based on the bovine isoform) and the numerous kinases implicated in the phosphorylation processes include AMP activated protein kinase (AMPK)^7^, protein kinase A (PKA)^8^, protein kinase B (PKB/Akt)^9^, protein kinase C (PKC)^10^, Ca^2+^-calmodulin-dependent kinase II^11^ and RhoA-activated kinase (ROCK)^12^. The influence of phosphorylation of the above sites on the activity of eNOS is under continuous evaluation and includes certain controversies^4^,^5^,^6^. Even though, it is generally accepted that increase in the level of phosphorylated Ser617 (eNOS^pSer617^), Ser635 (eNOS^pSer635^) and Ser1179 (eNOS^pSer1179^) results in activation of eNOS, whereas enhanced phosphorylation of Ser116 (eNOS^pSer116^) or Thr497 (eNOS^pThr497^) has inhibitory impacts^6^. The kinases that phosphorylate eNOS at the distinct sites are quite well characterized, but less is known about the protein phosphatases which catalyze the dephosphorylation processes. Protein phosphatase-1 (PP1), −2A (PP2A) and −2B (PP2B, also termed calcineurin) are implicated in the dephosphorylation of eNOS and certain site-specificity of these phosphatases has also been claimed in previous reports. Thus, it is suggested that PP2B^13^ and/or PP2A^14^ dephosphorylate eNOS^pSer116^, PP1^10^ and/or PP2B^15^ act on eNOS^pThr497^ and PP2A^10^,^16^ may be involved in eNOS^pSer1179^ dephosphorylation. Both PP1 and PP2A exist in the cells in various holoenzyme forms, however, still limited amount of data available on which PP1 or PP2A species are involved in the dephosphorylation of eNOS at the above phosphosites.

Phosphorylation/dephosphorylation of Thr497 appears to be a major factor in the regulation of eNOS activity. It is established that increase in the intracellular Ca^2+^ concentration contributes to eNOS activation^17^ as eNOS, to be fully active, requires binding of Ca^2+^-calmodulin (CaM) to a linker sequence separating the N- and C-terminal domains^18^. Increase in the level of eNOS^pThr497^ in endothelial cells in a PKC- or ROCK-dependent manner^19^, opposes CaM-binding leading to reduction in eNOS activity^11^. It is believed that one of the important determinants in eNOS activity is the reciprocal phosphorylation of Thr497 and Ser1179, respectively, implying that during agonist stimulation of endothelial cells (ECs) dephosphorylation of eNOS^pThr497^ precedes phosphorylation at Ser1179^10^,^11^,^15^. Accordingly, in bradykinin stimulated ECs activation of eNOS primarily involves dephosphorylation of eNOS^pThr497^ and the contribution of increased eNOS^pSer1179^ is assumed to be less significant^11^. It has also been proposed that eNOS^Thr497^ phosphorylation may not exclusively act via suppressing CaM-binding only, but it might serve also as an intrinsic switch in determining whether eNOS generates NO or superoxide in cells^20^.

Despite the crucial importance of the phosphorylation/dephosphorylation of Thr497 in eNOS activity and functions, the phosphatase holoenzyme(s) as well as the signalling mechanism implicated in eNOS^pThr497^ dephosphorylation have remained to be elucidated. Several reports^10^,^11^,^21^ have established that PP1 type enzyme dephosphorylates eNOS^pThr497^, however, the mediatory role (direct or indirect) of PP2A or the CaM-dependent PP2B/calcineurin could not be excluded either^15^.

The aim of the present study was to investigate if myosin phosphatase (MP) holoenzyme consisting of PP1 catalytic subunit (PP1c), myosin phosphatase target subunit-1 (MYPT1) and a 20 kDa protein in smooth muscles^22^,^23^, is involved in the dephosphorylation of eNOS^pThr497^. It is proven that the MP holoenzyme is an eNOS^pThr497^ phosphatase and MYPT1 targets PP1c to eNOS^pThr497^ substrate during dephosphorylation. Moreover, phosphorylation/dephosphorylation of MYPT1 at the inhibitory Thr696 residue parallels with changes in the eNOS^pThr497^ level confirming the involvement of MP in eNOS^Thr497^ dephosphorylation. We provide evidence for the interplay of MP and PP2A in the dephosphorylation of eNOS in ECs. Epigallocatechin-3-gallate (EGCG), a green tea polyphenol, signals ECs via the 67-kDa laminin receptor (67LR) to activate a specific isoform of trimer PP2A in a PKA dependent manner. The activated PP2A causes stimulation of MP through PP2A-induced dephosphorylation of MYPT1^pThr696^ which results in subsequent dephosphorylation of eNOS^pThr497^ and the 20 kDa light chain of myosin II (MLC20) coupled to increased eNOS activity and NO production as well as improvement of the barrier function of ECs.

## Results

### Interaction of eNOS with myosin phosphatase target subunit-1 (MYPT1)

It is generally accepted that most of the regulatory subunits in PP1 holoenzymes have a targeting role implying that they also interact with substrates and help PP1c to fulfil its catalytic action during the dephosphorylation process. In case of MP uncovering of MYPT1 binding protein partners led to the identification of several putative substrates^24^ and other interacting proteins important in the regulation of MP activity^25^. Thus, to prove eNOS as a possible substrate of MP we assessed initially if eNOS and MYPT1 interact in ECs or in tsA201 cells where these two proteins were co-expressed.

**Figure 1:**
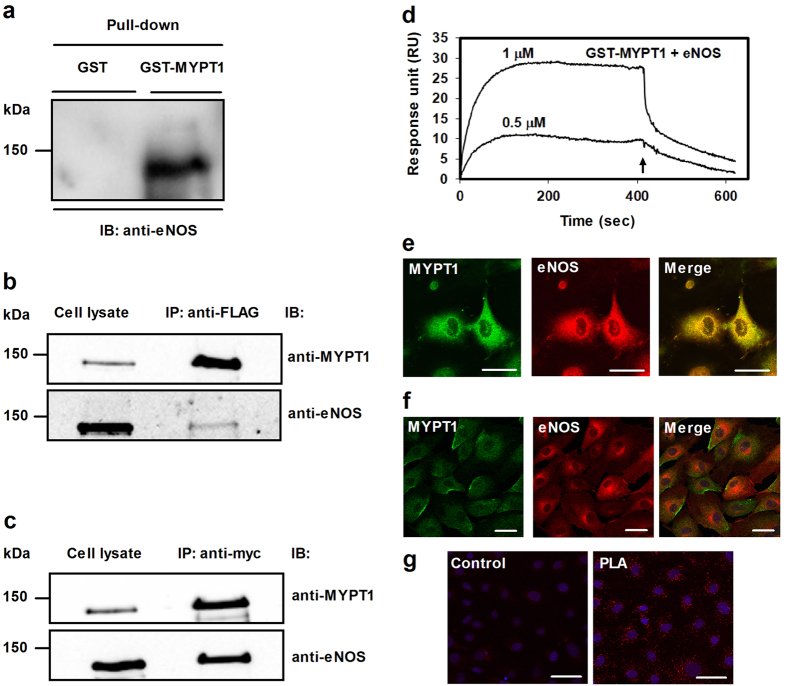
Interaction of eNOS with MYPT1. (**a**) Identification of eNOS in GST (control) or GST-MYPT1 pull-down fractions of HUVEC lysate using glutathione-Sepharose to isolate the interacting proteins. (**b**,**c**) Co-immunoprecipitation of overexpressed FLAG-MYPT1 and c-myc-eNOS in tsA201 cell lysates using anti-FLAG (**b**) or anti-c-myc-agarose (**c**) beads to isolate the protein complexes. Cropped images of representative Western blots are shown in (**a**,**b**) and (**c**). Uncropped, full-length blots are presented in Supplementary Information in Fig. S1. (**d**) Binding of eNOS to full-length GST-MYPT1 immobilized to anti-GST coupled CM5 sensor chips. Purified c-myc-eNOS at 0.5 or 1 μM concentrations in running buffer (10 mM Hepes (pH 7.4), 0.15 M NaCl, 3 mM EDTA, and 0.005% (v/v) Surfactant P20) was injected over the GST (control) and GST-MYPT1 surfaces at 0 time and the association phase of the interaction was monitored for 7 min. The dissociation phase in running buffer without c-myc-eNOS was started (at 7 min as indicated by an arrow) and recorded for 3.5 min. The surface (with immobilized recombinant GST) was treated identically to the GST-MYPT1 surfaces to determine unspecific binding which was subtracted from the data obtained with the GST-MYPT1 surfaces. Representative sensorgram of two independent experiments are shown. (**e**) Co-localization of MYPT1 (green) and eNOS (red) in human pulmonary artery endothelial cells (HPAEC). Images were captured by confocal microscopy. Merged images of eNOS and MYPT1 are also shown. Scale bar, 10 μm. (**f**) Co-localization of MYPT1 (green) and eNOS (red) in BPAECs. Images were captured by confocal microscopy and merged images are shown. Scale bar, 50 μm. (**g**) MYPT1 and eNOS interactions were assessed by Duolink proximity ligation assay (PLA) as described in Materials and Methods. For the control only the secondary antibodies were added. Red fluorescence indicates interacting MYPT1-eNOS complexes. Scale bar, 50 μm.

First, pull-down of eNOS from human umbilical vein EC (HUVEC) lysate by GST-MYPT1 was attempted capturing recombinant GST-MYPT1 and the associated endothelial proteins on glutathione-Sepharose (Fig. 1a). On Western blots eNOS was identified in the eluate using anti-eNOS suggesting its co-precipitation with GST-MYPT1, whereas no eNOS was detected in the control GST-pull-down fraction. Pull-downs from the lysates of tsA201 cells co-expressing both FLAG-MYPT1 and c-myc-eNOS proteins were analysed with anti-MYPT1^1–296^ and anti-eNOS (Fig. 1b and c), or with anti-FLAG or anti-c-myc antibodies (Fig. S1b) and these data confirmed reciprocal co-precipitation of c-myc-eNOS and FLAG-MYPT1 with each other. The whole blot images of GST- or GST-MYPT1 pull-downs, the expression pattern of FLAG-MYPT1 and myc-eNOS in tsA201 cells as well as control pull-downs from nontransfected tsA201 lysates with anti-FLAG and anti-c-myc antibodies are shown in Fig. S1.

C-myc-eNOS was purified from the lysate of c-myc-eNOS overexpressing tsA201 cells using anti-c-myc affinity matrix. This purified eNOS was applied as analyte in surface plasmon resonance (SPR) based binding assays to assess its interaction with GST-MYPT1 (Fig. 1d) immobilized on anti-GST coupled sensor chip^25^. The sensorgrams obtained at two different eNOS concentrations indicated stable interaction of MYPT1 with eNOS.

Experiments to visualize possible interactions of eNOS and MYPT1 *in situ* in ECs of different origin were carried out using confocal microscopy. Co-localization of eNOS and MYPT1 was apparent at the perinuclear regions in human pulmonary artery EC (HPAEC) (Fig. 1e). Co-localization of MYPT1 and eNOS was also identified in bovine pulmonary artery EC (BPAEC) (Fig. 1f) at similar locations to that of HPAECs and confirmed by proximity ligation assay, too (Fig. 1g). Thus, the above data suggest that eNOS and MYPT1 interact in ECs implying that MP may be a possible PP1 holoenzyme candidate involved in the dephosphorylation of eNOS.

**Figure 2:**
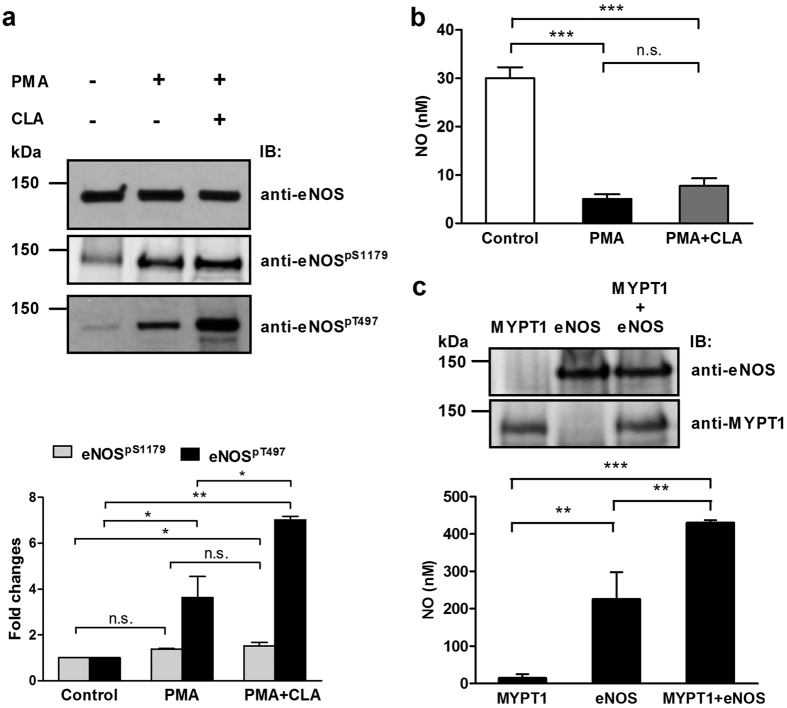
Phosphorylation of eNOS in eNOS expressing tsA201 cells upon challenges with PMA, or PMA plus CLA. (**a**) Phosphorylation of eNOS at Ser1179 or at Thr497 was analysed using anti-eNOS^pSer1179^ or anti-eNOS^pThr497^ in untreated control, and in 100 nM PMA (for 30 minutes) or 10 nM CLA plus 100 nM PMA (for 30 minutes) treated cells. Anti-eNOS was applied to determine eNOS as a loading control. Representative Western blots (upper panel) and densitometric analysis of the blots (lower panel) of three independent experiments are shown. (**b**) Effect of 100 nM PMA (for 30 minutes) or 10 nM CLA plus 100 nM PMA (for 30 minutes) treatments on NO production in the culture medium of eNOS overexpressing tsA201 cells. Thirty minutes following application of the effectors NO was determined as described in Materials and Methods. (**c**) The effect of co-expression of MYPT1 and eNOS in tsA201 cells on eNOS activity and NO production. Approximately equal number of tsA201 cells were plated in 6 well plates and 24 hours later the cells were transfected with pM11-FLAG-MYPT1 and/or pcDNA3.1c-myc-eNOS. 48 hours after transfection NO was determined in the culture medium. Data represent means ± SEM (n = 3), n.s.: not significant, *p  < 0.05, **p < 0.01, ***p < 0.001, One-way ANOVA, Newman-Keuls post-hoc testing. Cropped images of representative Western blots are shown in (**a**) and (**c**). Uncropped, full-length blots are presented in Supplementary Information in Fig. S8.

The tsA201 cells do not express eNOS, however, they are assumed to include the kinase/phosphatase machineries able to phosphorylate/dephosphorylate eNOS and MYPT1^25^. Thus, tsA201 cells transfected with c-myc-eNOS construct appeared to be a suitable model system to assess phosphorylation/dephosphorylation of eNOS upon PKC activation and phosphatase inhibition by calyculin-A (CLA), a cell permeable toxin inhibitor of PP1 and PP2A. Figure 2a shows that in c-myc-eNOS expressing cells there is a basal level of phosphorylation at Ser1179 of eNOS (eNOS^pSer1179^), while eNOS^pThr497^ is very low (Fig. 2a, upper panel). In agreement with the low level of the inhibitory phosphorylation (eNOS^pThr497^) and the occurrence of activatory phosphorylation (eNOS^pSer1179^) eNOS expressing tsA201 cells produced significant amount of NO as judged by NO measurements (Fig. 2b). Activation of PKC by treatment of tsA201 cells with phorbol 12-myristate-13-acetate (PMA) alone, or in combination with CLA, increased the level of eNOS^pThr497^ by ~4-fold or ~7-fold, respectively. On the other hand the level of eNOS^pSer1179^ was slightly enhanced only. PMA, or PMA plus CLA treatments suppressed dramatically the NO production of eNOS expressing tsA201 cells as shown on Fig. 2b. These data confirm previous suggestions that phosphorylation of eNOS at Thr497 is a major determinant in the regulation of eNOS activity^11^.

We also tested how FLAG-MYPT1 overexpression may influence eNOS activity in eNOS expressing tsA201 cells. As seen in Fig. 2c, expressing FLAG-MYPT1 alone did not induce NO production. When c-myc-eNOS was expressed alone NO synthesis was observed which was significantly higher when FLAG-MYPT1 was co-expressed with c-myc-eNOS in tsA201 cells. These data might also implicate MYPT1 in the regulation of eNOS activity and NO production. To further confirm the above data we also carried out experiments with eNOS knock-in HEK293 cells (HEK293-eNOS) in which FLAG-MYPT1 was overexpressed, then NO production was measured in the absence or presence of N-nitro-L-arginine-methyl ester (L-NAME), an eNOS inhibitor (Fig. S2). NO production was higher in HEK293-eNOS cells compared to HEK293 cells. It was also apparent that FLAG-MYPT1 overexpression increased eNOS activity significantly, however, it was not due to changes either in eNOS expression or in the phosphorylation level of eNOS^pThr497^ (Fig. S2, upper panel). L-NAME suppressed increased eNOS activity to control level in either the untransfected or FLAG-MYPT1 transfected HEK293-eNOS cells (Fig. S2, lower panel).

Other posttranslational modifications of eNOS such as phosphorylation at tyrosine657 (Tyr657)^26^ or S-glutathionylation at cysteine residues^27^ have also been reported to decrease eNOS activity. We observed no differences in eNOS phosphorylation on Tyr657 in eNOS knock-in HEK293 cells, which however might be due to the limited specificity of the used commercial antibody for this modification (not documented). Similarly, the immunoprecipitated S-glutathionylated proteins from eNOS knock-in HEK293 cells were limited and among them eNOS was not identified. Based on these results we conclude that Tyr657 phosphorylation or S-glutathionylation plays no role in the regulation of eNOS activity in our cell culture model.

### Identification of MP as an eNOS^pThr497^ phosphatase

**Figure 3:**
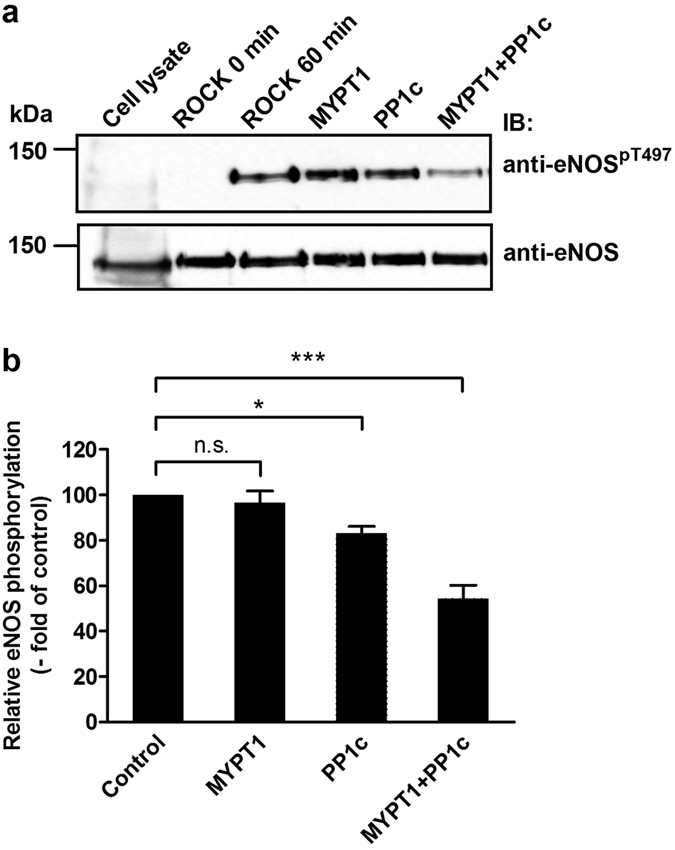
Myosin phosphatase dephosphorylates eNOS at the inhibitory phosphorylation site, (pThr497): the role of PP1c and MYPT1 in the dephosphorylation of eNOS^pThr497^. (**a**) c-myc-eNOS was isolated from the lysate of c-myc-eNOS expressing tsA201 cells on anti-c-myc-agarose beads and was phosphorylated by Rho-kinase (ROCK) for 60 minutes as described in Materials and Methods. Then, the resin was separated into four equal parts. Phosphorylated c-myc-eNOS was subjected to dephosphorylation for 15 min by FLAG-MYPT1 alone, PP1c alone, or by a mixture of PP1c and FLAG-MYPT1. The relative level of eNOS^pT497^ on anti- eNOS^pT497^ developed blots after phosphorylation by ROCK but before addition of the phosphatase components was considered as control (100%). Cropped images of representative Western blots are shown in (**a**). Uncropped, full-length blots are presented in Supplementary Information in Fig. S8. Densitometric analysis (**b**) of blots from three independent experiments are shown (means ± SEM (n = 3), *p < 0.05, **p < 0.01, One-way ANOVA, Newman-Keuls post-hoc testing).

C-myc-eNOS was expressed in tsA201 cells and isolated on anti-c-myc-Sepharose, then the matrix-bound eNOS was phosphorylated by ROCK at Thr497 (Fig. 3a) in the presence of microcystin-LR (PP1 and PP2A inhibitor). After washing the anti-c-myc-Sepharose bound phosphorylated eNOS to remove ATP, Mg^2+^ and microcystin-LR, the mixture was divided into four equal aliquots and beside control (incubated with assay buffer only) they were subjected to addition of FLAG-MYPT1, PP1c and PP1c plus FLAG-MYPT1. It is shown that FLAG-MYPT1 was without effect, while PP1c alone dephosphorylated eNOS^pThr497^ to a relatively low extent, which was significantly increased when both PP1c and FLAG-MYPT1 were present (Fig. 3a and b). These results suggest that MP holoenzyme (i.e. the PP1c-MYPT1 complex) is an eNOS^pThr497^ phosphatase, and since MYPT1 enhances the phosphatase activity of PP1c on eNOS^pThr497^ it fulfils a targeting and activating role in this dephosphorylation process.

**Figure 4:**
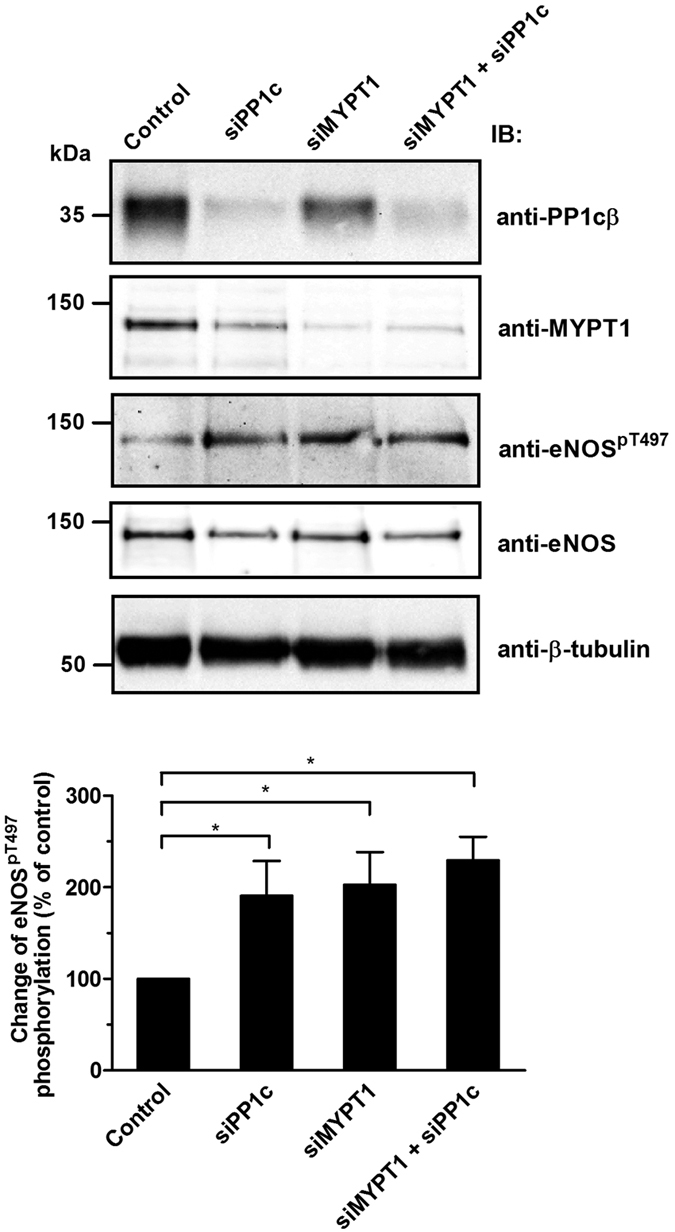
Effect of silencing of PP1c, MYPT1, or both PP1c and MYPT1 by siRNAs on the level of eNOS^pThr497^. BPAECs were transfected with nonspecific (scrambled) siRNA (control), or with siRNA-PP1, siRNA-MYPT1 and with a mixture of siRNA-PP1 and siRNA-MYPT1. The changes of PP1cδ and MYPT1 protein levels, as well as the level of eNOS^pThr497^ was determined by Western blotting. Cropped images of representative Western blots are shown (upper panel). Uncropped, full-length blots are presented in Supplementary Information in Fig. S8. The bar graph represents the change in the level of eNOS^pThr497^ compared to the control. Densitometric analysis of blots from at least four independent experiments (lower panel) are shown (means ± SEM, *p < 0.05, One-way ANOVA, Newman-Keuls post-hoc testing).

To confirm further the role of PP1c and MYPT1 in the dephosphorylation of eNOS^pThr497^ silencing of these proteins in BPAECs with siRNAs were carried out and in parallel the level of eNOS^pThr497^ was also determined. PP1c was silenced with siRNA specific for all isoform (PP1cα, PP1cβ/δ, PP1cγ1), however decrease in the level of PP1cδ was assessed as this isoform was identified to associate with MYPT1specifically in cells^28^. It is apparent on Fig. 4 (upper panel) that silencing either PP1cδ or MYPT1, or both, led to decreased expression of these proteins (see Fig. S3 for the statistics of silencing efficiency) which was accompanied by an increase in the level of eNOS^pThr497^ (Fig. 4, upper and lower panels) implicating both subunits of MP holoenzyme in the dephosphorylation of this phosphorylated residue.

### The effects of PKC activation and phosphatase inhibition on the inhibitory phosphorylation of eNOS and MYPT1

**Figure 5:**
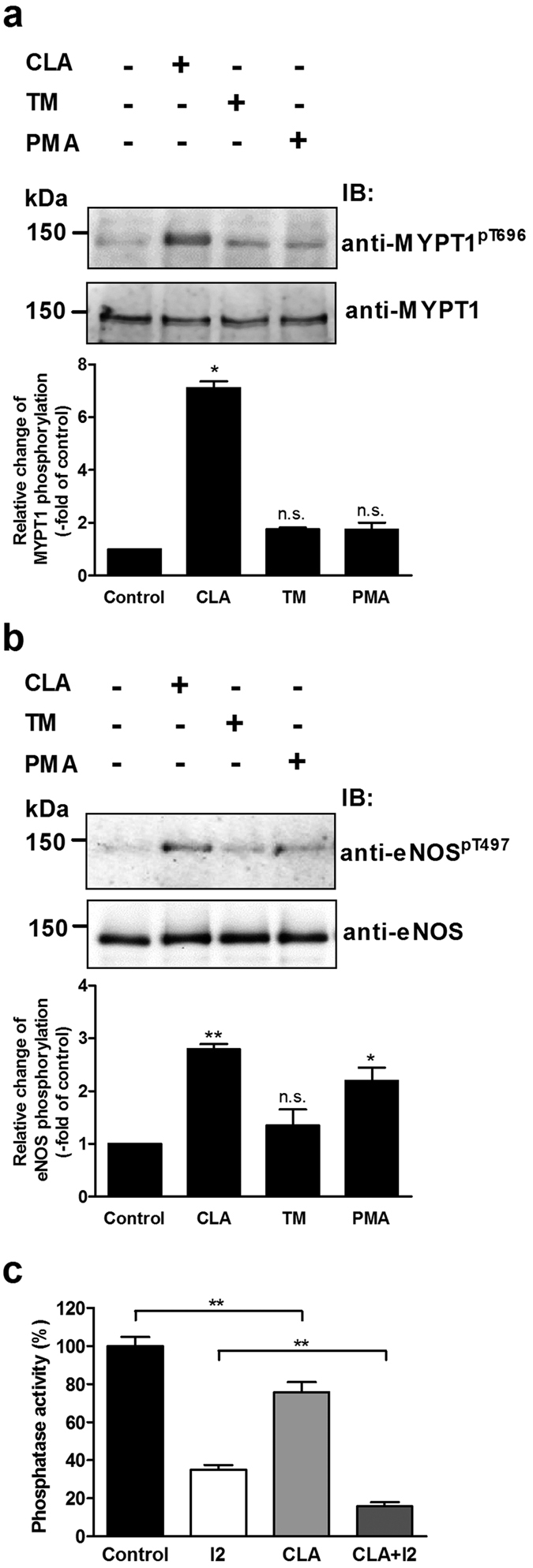
The effect of PKC activation and phosphatase inhibition on the level of MYPT1^pThr696^ and eNOS^pThr497^. BPAECs cells were treated with 10 nM CLA, 1 μM TM or 100 nM PMA for 30 min and the level of MYPT1^pThr696^ (**a**) and eNOS^pThr497^ (**b**) was monitored by Western blotting (upper panel) and quantified by densitometry (bar graphs). Cropped images of representative Western blots are shown in (**a**) and (**b**). Uncropped, full-length blots are presented in Supplementary Information in Fig. S8. Densitometric analysis of blots from 3–4 independent experiments was carried out (means ± SEM, n.s.: not significant, *p < 0.05, **p < 0.01, ***p < 0.001, compared to control. One-way ANOVA, Newman-Keuls post-hoc testing). (**c**) BPAECs cells were treated with 10 nM CLA and the phosphatase activity in the lysates of untreated (control) or CLA treated cells was determined in the absence or the presence of 2 μM inhibitor-2 using ^32^P-MLC20 as substrate. Data represent means ± SEM, **p < 0.01 (n = 3), One-way ANOVA, Newman-Keuls post-hoc testing.

We challenged BPAECs with 10 nM calyculin-A (CLA) or 1 μM tautomycin (TM) to inhibit PP2A and PP1, respectively. CLA and TM inhibit both PP2A and PP1 in *in vitro* phosphatase assays and there are also controversies concerning their selectivity in cellular systems^29^,^30^. However, our recent results^31^ confirmed that CLA (up to 50 nM) caused partial, but predominant inhibition of PP2A, while TM (up to 1 μM) primarily suppressed PP1 in THP-1 cells. Figure 5a illustrates that treatment of BPAECs with CLA alone resulted in a dramatic increase in the phosphorylation of MYPT1 at Thr696 (MYPT1^pThr696^) while TM and PMA did not increase significantly the level of MYPT1^pThr696^. Figure 5b illustrates that enhanced level of eNOS^pThr497^ paralleled with the increase of the inhibitory phosphorylation of MP in MYPT1 (MYPT1^pThr696^). In contrast, TM increased the level of eNOS^pThr497^ slightly, but not significantly. Activation of PKC by PMA also increased the level of eNOS^pThr497^ in accordance with results shown earlier for eNOS expressing tsA201 cells (see Fig. 2a). As PP2A is assumed to dephosphorylate MYPT1^pThr696^, the above data support the scenario that PP2A specific inhibition increased the level of MYPT1^pThr696^ resulting in inhibition of MP and suppression of eNOS activity as a consequence of increase in eNOS^pThr497^. We determined the distribution of the activity of PP1 and PP2A in the lysates of untreated or CLA treated BPAECs using inhibitor-2 (I2) to specifically inhibit PP1 and attempted also to identify type specific inhibition by CLA (Fig. 5c). It is seen that ~65% of the total phosphatase activity was due to PP1 while PP2A (the activity measured in the presence of I2) represented ~35%. CLA decreased the phosphatase activity of the lysate by ~25%, and a large portion (~20%) of this inhibitory impact was exerted on the I2 suppressed (i.e. PP2A) activity indicating predominant inhibition of PP2A by CLA in accord with our previous findings^31^ in THP-1 cells.

**Figure 6:**
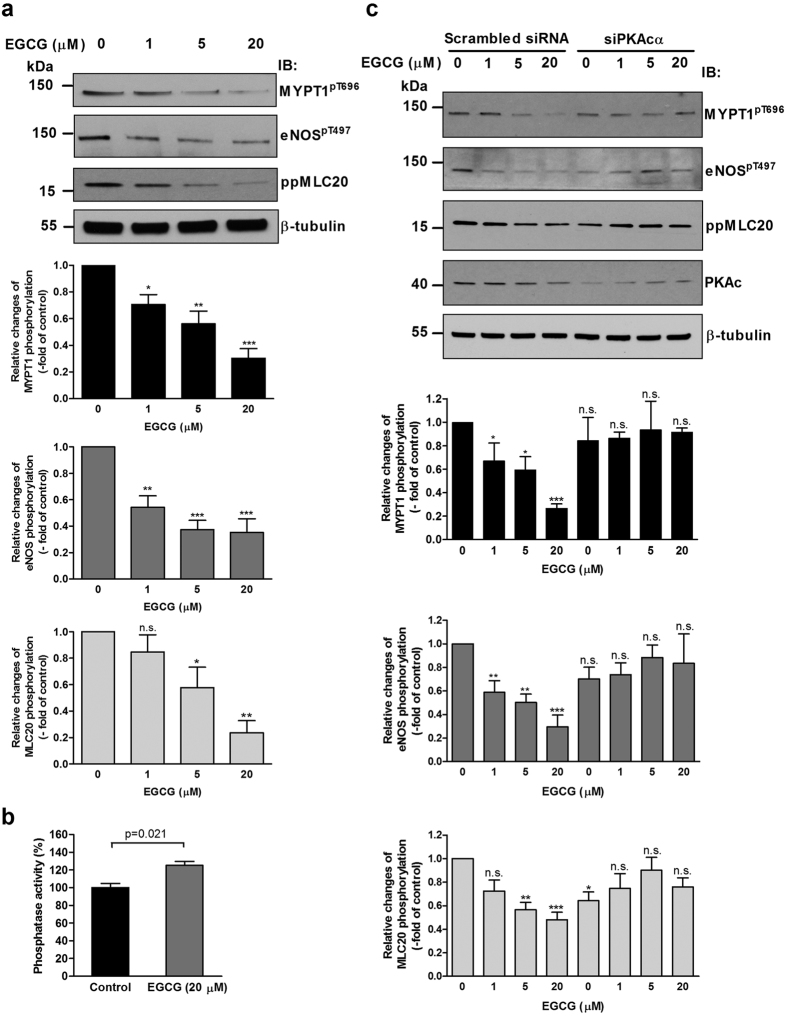
The effect of EGCG treatment and PKA silencing on the level of MYPT1^pThr696^, eNOS^pThr497^ and ppMLC20^Thr18/Ser19^. (**a**) BPAECs were pretreated with 100 nM PMA for 30 minutes, then challenged with EGCG in the indicated concentrations for 1 hour. Changes in the level of MYPT1^pThr696^, eNOS^pThr497^ and ppMLC20 were assessed by Western blotting with phospho-specific antibodies upon the different challenges (upper panel). Cropped images of representative Western blots are shown. Uncropped, full-length blots are presented in Supplementary Information in Fig. S8. Bar graphs represent the changes in the level of MYPT1^pThr696^ (upper graph), eNOS^pThr497^ (middle graph) and ppMLC20 (lower graph) determined by densitometric analysis of blots from 3–4 independent experiments (means ± SEM, n.s.: not significant, *p < 0.05, **p < 0.01, ***p < 0.001 compared to control, i.e. without EGCG, One-way ANOVA, Newman-Keuls post-hoc testing). (**b**) BPAECs cells were treated with 20 μM EGCG and the phosphatase activity in the lysates of untreated (control) or EGCG treated cells was determined using ^32^P-MLC20 as substrate. Data represent means ± SEM (n = 3), two-tailed Student’s *t*-test. (**c**) BPAECs were transfected with scrambled or PKA catalytic subunit (PKAc) specific siRNA then treated with PMA and EGCG and the changes in the level of MYPT1^pThr696^, eNOS^pThr497^ and ppMLC20 were assessed as described in (**a**).

The above data are consistent with a dephosphorylation sequence of PP2A → MYPT1^pThr696^-PP1c → eNOS^pThr497^, but this putative mechanism requires further experimental confirmation. Therefore, activation of PP2A in BPAECs and subsequent dephosphorylation of both MYPT1^pThr696^ and eNOS^pThr497^ were probed. It was previously shown that in melanoma cells EGCG binds to the 67LR^32^ and increases PP2A activity^33^ in a protein kinase A (PKA) dependent manner. This activation of PP2A was shown earlier to be specific for the PP2A trimer holoenzyme including the Bδ subunit and was due to phosphorylation of Bδ by protein kinase A (PKA)^34^. Both 67LR and PP2A-Bδ (see Fig. S4) as well as PKA (see Fig. 6c) are present in BPAECs, therefore activation of PP2A with EGCG was attempted. Since the endogenous level of eNOS^pThr497^ in BPAECs was quite low, we treated the cells with PMA to achive a higher level of eNOS^pThr497^ in order to detect more reliably eNOS^pThr497^ dephosphorylation. Treatment of BPAECs with EGCG in a concentration range from 1 μM to 20 μM resulted in substantial decrease in the levels of both MYPT1^pThr696^ (Fig. 6a, upper panel and upper bar graph) and eNOS^pThr497^ (Fig. 6a, upper panel and middle bar graph), suggesting that PP2A is involved in their dephosphorylation. Presumably, PP2A induces activation of MP via dephosphorylation of MYPT1^pThr696^ resulting in subsequent dephosphorylation and activation of eNOS as well. Moreover, EGCG also induced substantial dephosphorylation of 20 kDa light chain of myosin II (MLC20) as assessed by an antibody specific for diphosphorylated MLC20 (ppMLC20) as shown in Fig. 6a (upper panel and lower bar graph). In accordance with the above assumptions Fig. 6b demonstrates that EGCG treatment of BPAECs significantly increased the phosphatase activity. It is to note that EGCG has been shown to increase the phosphorylation level at Ser1179 of eNOS, a phosphosite which is known to stimulate eNOS activity^35^,^36^,^37^. We found that under our experimental conditions (i.e. when BPAECs were pretreated with PMA) there was a substantial basal phosphorylation level at Ser1179 of eNOS and a slight increase upon EGCG treatment was observed only at 5 μM EGCG (Fig. S5).

Next, we searched for further evidence for the PKA dependence of the EGCG induced dephosphorylation processes. We silenced the catalytic subunit α of PKA (PKAcα) in BPAECs and assessed the level of MYPT1^pThr696^, eNOS^pThr497^ and ppMLC20 in the cells. Silencing efficacy and statistics are shown in Fig. S6. Figure 6c illustrates the changes in the level of MYPT1^pThr696^, eNOS^pThr497^ and ppMLC20 in BPAECs transfected with scrambled or PKAcα specific siRNAs and then challenged with different concentration of EGCG. It is apparent that transfection of BPAECs with scrambled siRNA resulted in similar patterns of dephosphorylation of MYPT1^pThr696^, eNOS^pThr497^ and ppMLC20 upon EGCG treatments as observed in Fig. 6a. In BPAECs transfected with PKAcα specific siRNAs a slight decrease in the basal phosphorylation of MYPT1^pThr696^, eNOS^pThr497^ and ppMLC20 was observed, however, no significant dephosphorylation of these proteins were detected upon challenges by EGCG. These data confirmed that EGCG induced phosphatase activation in BPAECs is also accomplished in a PKA dependent manner.

### The effects of PKC activation and phosphatase inhibition/activation on NO production and transendothelial electrical resistance of BPAECs

**Figure 7:**
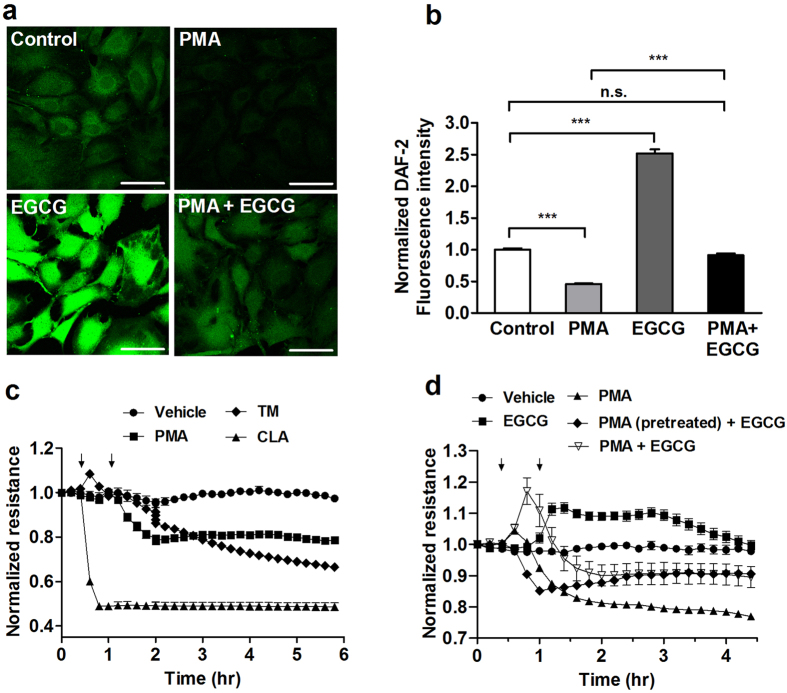
Effect of PKC activation and phosphatase inhibition/activation on NO production and the transendothelial electrical resistance (TER) of BPAECs. (**a**) BPAECs were grown on coverslips in serum free medium and loaded with the reaction mixture including DAF-2 DA for 60 min. Next, coverslips were incubated with the PKC activator PMA (100 nM), EGCG (20 μM) and the combination of PMA and EGCG in parallel with the non-treated coverslips (control) for 60 min. Scale bar, 50 μm. (**b**) Single cell fluorescence activity determined by ImageJ after different treatments indicated in (**a)**. Results of single cell values (120–153 individual cells) of two independent experiments are shown in means ± SEM. (n.s.: not significant, ***p < 0.001, One-way ANOVA, Newman-Keuls post-hoc testing). (**c**) BPAECs grown in serum free medium in ECIS arrays were untreated (control), or treated with PMA (100 nM), 10 nM CLA or with 1 μM tautomycin (TM) for 30 minutes. (**d**) BPAECs cells grown on ECIS arrays were treated with vehicle, 20 μM EGCG, 100 nM PMA or with 100 nM PMA and 20 μM EGCG, added together or EGCG was added 30 minutes after PMA. Time points of treatment with effectors are indicated by arrows. Normalized TER values were expressed as mean ± SEM of 3–4 individual experiments.

The effect of PKC activation as well as phosphatase inhibition/activation was also assayed on physiological responses of BPAECs such as NO production and barrier function. The latter was characterized by measuring transendothelial electrical resistance (TER) (Fig. 7c and d). For NO measurement NO specific 4,5-diaminofluorescein diacetate (DAF-2 DA) fluorescence of cells was captured (Fig. 7a). BPAECs loaded with the reaction mixture including DAF-2 DA were subjected to different treatments, then fluorescent intensities of cells were determined (Fig. 7b). It is seen that PMA moderately, but significantly decreased NO synthesis. In contrast, EGCG spectacularly enhanced NO production and it also attenuated PMA suppressed NO synthesis. These changes in NO synthesis appear to correlate well with how these effectors influenced the level of eNOS^pThr497^ (see Figs 5 and 6a,b). It was also established that the increase in NO of BPAECs challenged by either acetylcholine or EGCG was profoundly inhibited when the cells were preincubated with L-NAME (Fig. S7).

In another set of experiments we investigated how PMA, CLA, TM, EGCG and the combination of PMA and EGCG affect TER of BPAECs. PMA and TM suppressed TER moderately and the decreasing tendency in TER appeared to be partially reversible in case of PMA, but irreversible with TM during the assay period (Fig. 7c). CLA also induced a fast and dramatic decrease in TER in an irreversible manner. In accord with our earlier findings^25^ this “acute” effect of the phosphatase inhibitors on TER was presumably due to the inhibition of MP resulting in sustained phosphorylation of MLC20 which contributes highly to decreasing TER. In contrast, EGCG enhanced TER of BPAECs and attenuated suppression of TER by PMA either when it was added together or 30 min after PMA (Fig. 7d). These data suggest that PKC activation or phosphatase inhibition suppress TER compromising barrier function of BPAECs. On the other hand, phosphatase activation by EGCG appears to improve barrier function and helps to restore, at least in part, the effect of barrier suppressing agents.

## Discussion

Phosphorylation/dephosporylation of eNOS at Thr497 is a crucial factor in the mediation of eNOS activity and it is also involved in balancing whether eNOS generates NO or superoxide^20^. PKC and ROCK are the kinase candidates to target Thr497 under physiological conditions^19^, while the dephosphorylating phosphatase is not unambiguously identified. There appears to be a consensus in the literature that PP1 type phosphatase acts on phosphorylated Thr497 in eNOS (eNOS^pThr497^) in ECs^10^,^11^,^21^. Surprisingly, the type of PP1 holoenzyme has not been identified yet, although this knowledge is essential to uncover physiological regulatory events in the dephosphorylation of eNOS^pThr497^. Our present study identifies myosin phosphatase (MP), consisting of associated PP1cδ and MYPT1, as the phosphatase holoenzyme which dephosphorylates eNOS^pThr497^. This claim is supported by the following experimental data: (i) MYPT1 and eNOS co-precipitated from the lysates of HUVEC, HPAEC and BPAEC and their colocalization was also apparent in these ECs (see Fig. 1) (ii) SPR-based binding assays using purified proteins also confirmed stable interaction between c-myc-eNOS and GST-MYPT1 (iii) the PP1δ–MYPT1 complex dephosphorylated eNOS^pThr497^ effectively in which MYPT1 had a targeting role increasing the activity of PP1cδ toward eNOS^pThr497^ (see Fig. 3) (iv) silencing PP1δ, MYPT1 or both in BPAECs increased the level of eNOS^pThr497^ (see Fig. 4). We also provide evidence that overexpression of MYPT1 in eNOS expressing tsA201 cells or in eNOS knock-in HEK293 cells increased eNOS activity without changing the expression level of eNOS. This activation of eNOS could not be due to changes in eNOS^pThr497^ dephosphorylation either as in HEK293-eNOS cells eNOS^pThr497^ was quite low and did not change upon FLAG-MYPT1 overexpression (see Fig. S2, upper panel). We speculate that the stable interaction between MYPT1 and eNOS might influence intracellular localization of eNOS which could also affect enzyme activity^3^. It is to note, however, that in light of a recent report^38^ MYPT1 may also have an influence on eNOS expression as in basilar arteries of old, heterozygous MYPT1-Thr696Ala knock-in mice eNOS-mRNA was lower in knock-in compared to the wild-type animal. Experiments with this MYPT1-Thr696Ala knock-in mice also established the role of the phosphorylation of MYPT1^Thr696^ in the increased basal tone of basilar arteries. In summary, our present data implicates MP in the activation of eNOS accompanied by enhanced NO production in ECs. Thus, application of this regulatory scenario to smooth muscle tissues would imply that the NO diffused from ECs to smooth muscle cells may induce MP activation and relaxation of smooth muscles. Indeed, it was previously reported^39^ that the NO donor sodium nitroprusside (SNP) caused relaxation of histamine-contracted carotid media concurrent with activation of MP and with declined phosphorylation level of both MLC20 and the C-kinase activated phosphatase inhibitor of 17 kDa (CPI17), a phosphorylation dependent MP specific inhibitor protein. Moreover, SNP treatment triggered, beside a rapid dephosphorylation of CPI-17, a slow dephosphorylation of MYPT1 (both at phospho-Thr696 and phospho-Thr853) in denuded femoral artery suggesting an NO induced biphasic reactivation of MP by dephosphorylation during smooth muscle relaxation^40^. The extent of relaxation of precontracted arterial smooth muscle induced by endogenous NO released from the endothelium was similar to that of found with SNP treated denuded artery establishing the physiological significance and relevance of the above dephosphorylation processes in the mechanism of smooth muscle relaxation.

It has been established that regulation of MP activity occurs via inhibitors (toxins, inhibitory proteins) interacting with PP1cδ^22^,^23^,^31^ and/or by inhibitory phosphorylation of MYPT1 at Thr696 and Thr850 residues^41^. In this study regulation of eNOS^pThr497^ level by MP was modelled using cell permeable phosphatase inhibitory toxins (CLA and TM) at concentrations assumed to be specific for PP1 and PP2A^29^,^30^,^31^. It should be emphasized that the viability of ECs limits the applied toxin concentrations, therefore, only partial inhibition of PP2A or PP1 by these toxins could be assumed as we showed previously^31^. Our present data demonstrate that PP1 specific inhibition by TM increased eNOS^pThr497^ and MYPT1^pThr696^ moderately. In contrast, CLA enhanced both eNOS^pThr497^ and MYPT1^pThr696^ more profoundly via predominant inhibition of PP2A as validated by phosphatase assays of BPAEC lysates (Fig. 5b). These results are in accordance with previous findings demonstrating that inhibition of PP2A^41^ or PP2B^25^, enzymes which dephosphorylate MYPT1^pThr696^, leads to increased level of MYPT1^pThr696^. The latter is accompanied by MP inhibition and as a consequence enhanced phosphorylation level of MLC20, a dedicated substrate of MP. The above data establish a mechanism implying an interplay of PP2A and MP in the regulation of eNOS^pThr497^ dephosphorylation, however, the physiological significance of these events await further clarification.

**Figure 8:**
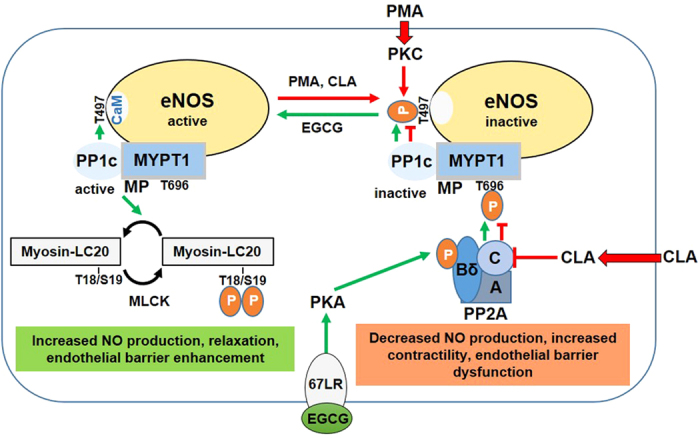
Regulation of eNOS activity and endothelial barrier function by phosphorylation-dephosphorylation. We have shown that myosin phosphatase (MP) interacts with eNOS via its myosin phosphatase target subunit-1 (MYPT1) which directs protein phosphatase-1 catalytic subunit (PP1c) for dephosphorylation of phospho-Thr497 allowing binding of Ca^2+^-calmodulin (CaM) and activation of eNOS. PMA and/or calyculin-A (CLA) increases the phosphorylation level of eNOS-Thr497 by activation of protein kinase C (PKC) and/or inhibition of PP2A, respectively (red lines). Suppression of PP2A activity results in increased phosphorylation of MYPT1 at Thr696 and inhibition of MP. Both PKC activation and MP inhibition contributes to the inactivation of eNOS via increased phosphorylation at Thr497 and thus interfering with CaM binding. Inactivation of eNOS accompanies by decreased NO production while the enhanced LC20 phosphorylation leads to increased contractility and thereby endothelial barrier dysfunction. Our present data suggest that the reactivation of eNOS and dephosphorylation of LC20 may be accomplished by an EGCG induced activation of both PP2A and MP involving the 67 kDa laminin receptor (67LR) and protein kinase A (PKA) in this phosphatase activation. The 67LR mediated action of EGCG implies activation of PKA which phosphorylates the Bδ subunit of PP2A resulting in PP2A activation and subsequent dephosphorylation of MYPT1 (at phospho-Thr696) and activation of MP (green lines). Then, the activated MP dephosphorylates both phosho-Thr497 in eNOS and phospho-Thr18/Ser19 in LC20 resulting in increased NO production and decreased contractility (relaxation) with endothelial barrier enhancement.

Previous work^10^ has shown that forskolin treatment of ECs stimulated dephosphorylation of eNOS^pThr497^ by PP1, suggesting the involvement of a signalling pathway via cAMP/PKA in PP1 activation, however, the detailed mechanism has not been elucidated. Our present results highlight another signalling mechanism for physiological stimulation of eNOS^pThr497^ dephosphorylation (see Fig. 8). This involves EGCG, a green tea polyphenol as an agonist interacting with 67LR and signalling downstream to activate PKA and PP2A. It has been proven in melanoma cell lines that EGCG stimulates the activity of PP2A via the 67LR in a cAMP/PKA-dependent manner and this activated PP2A dephosphorylated both phosphorylated CPI-17 and MYPT1^pThr696^ resulting in MP activation and dephosphorylation of merlin, another MP substrate^33^. Here, we confirmed that the major elements (67LR, PKA and PP2A-Bδ) required for this signalling are present in BPAECs (see Fig. S4), therefore, it is reasonable to assume that the EGCG → 67LR → PKA → PP2A pathway also operates in ECs and induces PP2A activation. It is apparent that PP2A activated along this pathway results in marked dephosphorylation of MYPT1^pThr696^ coupled with activation of MP (see Fig. 6) and subsequent dephosphorylation of eNOS^pThr497^ and ppMLC20 in ECs. The other side of the EGCG signalling is that it also results in increased level of phosphorylation at Ser1179 in eNOS (eNOS^pSer1179^)^35^,^36^,^37^ via the activation of PKA/phosphatidylinositol 3-kinase (PI3K)/protein kinase B (Akt) pathway^35^ and src-kinase^36^. In accordance with these findings EGCG also increased slightly the level of eNOS^pSer1179^ under our experimental conditions (Fig. 5S). Thus, the above data establish a physiologically relevant signalling pathway for the previously described reciprocal phosphorylation^10^,^11^,^15^ of eNOS at the inhibitory Thr497 and the activatory Ser1179 residues. Our present data support the existence of a novel pathway via EGCG → 67LR → PKA → PP2A → MP activation for effective dephosphorylation of both eNOS^pThr497^ and ppMLC20 (see Fig. 8).

EGCG and structurally related other catechins may have a plethora of intracellular targets and they may exert distinct physiological influences. In our previous work we showed that EGCG interacted with and inhibited PP1c activity at micromolar concentrations in *in vitro* phosphatase assays and binding of EGCG to the hydrophobic groove of the PP1c substrate binding region was also established^42^. However, only moderate extent of phosphatase inhibition occurred when intact cells were incubated with relatively high EGCG concentration (100–500 μM) and it was presumably due to low extent of EGCG permeation through the cell membranes^43^. These data suggest distinct mechanism of actions of EGCG on cells depending upon its applied concentrations. Thus, at low concentration (1–20 μM) EGCG exerts its effect through binding to 67LR initiating a signalling pathway leading to phosphatase activation. In contrast, at high concentration EGCG might also permeate cells to certain extent and binds to PP1c causing phosphatase inhibition. However, regarding the assumed EGCG concentration (~1 μM) after regular green tea consumption only the former process (EGCG → 67LR signalling) might have physiological relevance. Another issue is if other catechins structurally related to EGCG may exert similar effects on ECs. A study by Lorenz *et al*.^37^ reported that several catechins (epicatechin, epigallocatechin, epicatechin-3-gallate) which include structurally similar units present in EGCG were without significant effect on eNOS activity and NO production. On the contrary, Ramirez-Sanchez *et al*.^44^ demonstrated that (-)-epicatechin (EPI) activated eNOS and NO production in a stereospecific and Ca^2+^-independent manner in ECs. EPI enhanced the level of eNOS^pSer1179^ in accordance of its eNOS activating feature. However, with respect to its effect on eNOS^pThr497^ level controversial data have been published as upon EPI addition both decreased^45^ and unchanged^44^ level of eNOS^pThr497^ was reported. It has been suggested that EPI may exert its physiological effects via the pathway involving phosphatidylinositol 3-kinase (PI3K)^44^. However, it still remains to be elucidated if EPI acts via the same pathway(s) on ECs as does EGCG.

In agreement with previous studies^46^,^47^ we found that PKC activation by PMA, or phosphatase inhibition with CLA, caused decrease in TER of EC monolayers. The TER and NO suppressing effect of PMA was attenuated by EGCG which helped to restore, at least partially, barrier integrity and eNOS activity. There are apparent controversies with respect to how changes in NO level influence endothelial permeability (reviewed by Durán *et al*.^2^). It appears that hyperpermeability induced by inflammatory agents requires eNOS activation and increased NO production^48^. In contrast, our present findings are consistent with the conclusions that EGCG induced enhancement of NO is accompanied by stabilization and improvement of endothelial barrier function. These contradictory findings might be due to widespread influences of EGCG on the signalling pathways in ECs. On one hand, EGCG may also stimulate phosphorylation of Ser1179 in eNOS that is required to enhanced eNOS activity in ECs^35^,^36^,^37^ via a pathway distinct from that of operates during phosphatase activation. On the other hand, EGCG induced phosphatase activation is not limited only for enhanced dephosphorylation and stimulation of eNOS, but it also has a “relaxing” effect on the contractile machinery of ECs by inducing ppMLC20 dephosphorylation (see Fig. 6a and b) decreasing the amount of stress fibres and restoring gap formation between the cells. These events may also lead to decreased permeability and as a consequence barrier improvement of ECs. Moreover, this EGCG induced increase of NO was also observed in rat aortic rings^35^ or mesenteric vascular beds^36^ causing a dose- and endothelium dependent vasorelaxation of these smooth muscles. This vasodilation was suppressed by L-NAME indicating the direct role of eNOS in these events.

In summary, our present study contributes to the understanding of the regulation of eNOS activity in EC from an angle less frequently studied so far. Activation of eNOS requires dephosphorylation of eNOS^pThr497^, therefore, identification of the phosphatase involved may shed light on novel regulatory pathways. We have shown here that MP (the complex of PP1c and MYPT1) dephosphorylates eNOS^pThr497^ and the inhibitory phosphorylation of MYPT1 at Thr696 is a major regulatory event in eNOS dephosphorylation. The pathways regulating inhibitory MYPT1 phosphorylation have been described^22^,^23^, and among these signalling via RhoA/ROCK is involved in the phosphorylation of both Thr696 in MYPT1^25^ and Thr497 in eNOS^12^ in BPAECs. Thus, ROCK can act in a concerted manner on the phosphorylation/dephosphorylation of eNOS: it may phosphorylate Thr497 in eNOS^49^ and Thr696 in MYPT1 simultaneously leading to a sustained level of eNOS^pThr497^ and suppression of NO production. Another novel aspect of our present findings is that we identify a signalling pathway in ECs which operates via interaction of EGCG with 67LR leading to PP2A-driven activation of MP which accompanies with dephosphorylation (and activation) of eNOS and ppMLC20 (see Fig. 8). In addition to increasing eNOS activity and NO production EGCG also improves endothelial barrier function. It has been established that mono- and diphosphorylation of MLC20 is involved in the fine tuning of barrier disruption in ECs^50^. EGCG, by stimulating ppMLC20 dephosphorylation decreases the contractility of ECs thereby restoring and improving barrier function. Moreover, the NO synthesized in ECs plays crucial roles in the regulation of distinct cardiovascular events, therefore, uncovering the molecular background of the mediation of NO production may also have relevance with regards of cardiovascular pharmacology.

## Materials and Methods

### Materials

Anti-MYPT1^1–296^ (Lontay *et al*.)^51^, rabbit skeletal muscle PP1c and ^32^P-MLC20 (Tóth *et al*.)^52^ were prepared and characterized as described previously in the respective references. All other materials were obtained from commercial sources and given in Supplementary Information.

### Cell cultures

Umbilical cords were collected from the local hospital (Department of Obstretrics and Gynecology, University of Debrecen) during normal deliveries with informed consent given and the procedure was performed by the approval of the Ethics Committee of the University of Debrecen (Ref. number: 2909-2008) conform the Declaration of Helsinki. Human umbilical vein endothelial cells (HUVEC) were isolated as previously described (Palatka *et al*.)^53^. Cells were maintained in M199 media supplemented with 20% (v/v) fetal bovine serum (FBS), 10 mM HEPES, 2 mM L-glutamine, 0.25 μg/ml amphotericin B, 100 U/ml penicillin, 100 μg/ml streptomycin. Cells were cultured from the same batch and used at passages 4–7 for all experiments.

Bovine pulmonary artery endothelial cells (BPAEC) (cell culture line-CCL 209) were obtained frozen at passage 8 (American Type Tissue Culture Collection Rockville, MD, USA), and were used between passages 17–23. Cells were cultured in Minimum Essential Medium (MEM) supplemented with 10% (v/v) heat inactivated FBS, 1 mM sodium pyruvate, 0.1 mM non-essential amino acids solution, 2 mM glutamine, 1% (v/v) antibiotic-antimycotic solution. Human pulmonary artery endothelial cells (HPAEC) were purchased from Clonetics (San Diego, CA, USA) were cultured in EBM-2 supplemented with 10% (v/v) FBS, 0.2 ml of hydrocortisone, 2 ml of human FGF-B, 0.5 ml of VEGF, 0.5 ml of long-arm insulin-like growth factor-1 (R3-IGF-1), 0.5 ml of ascorbic acid, 0.5 ml of human epidermal growth factor (EGF), 0.5 ml of GA-1000, and 0.5 ml of heparin solutions. Cells were cultured from the same batch and used at passages 4–7 for all experiments (Kolozsvári *et al*.)^25^.

tsA201 (Health Protection Agency Culture Collection, Salisbury, UK), eNOS knock-in HEK293 cells (a kind gift of Dr. David Fulton, Augusta University, Augusta, GA, USA) and HEK293 cells (American Type Tissue Culture Collection Rockville, MD, USA) were cultured in Dulbecco’s modified Eagle’s medium (DMEM) supplemented with 10% (v/v) FBS, 2 mM L-glutamine, and 1% (v/v) antibiotic-antimycotic solution. Cells were maintained at 37 °C in a humidified atmosphere of 5% CO_2_.

### Immunoblotting

Preparation of the samples was as follows. After phosphatase inhibitor treatment the cells were lysed in 0.1% (v/v) Triton X-100,150 mM NaCl, 50 mM Tris–HCl (pH 7.4), 20 mM EDTA and 0.5% (v/v) protease inhibitor mix containing lysis buffer. For immunoprecipitation and pull-down assays the cells were lysed in 0.1% (v/v) Triton X-100, 150 mM NaCl, 50 mM Tris–HCl (pH 7.4), 20 mM EDTA and 0.5% (v/v) protease inhibitor mix containing buffer. All of the samples were boiled for 10 minutes at 100 °C in SDS sample buffer in a final concentration of 62.5 mM Tris-HCl (pH 6.8), 2% (m/v) SDS, 10% (v/v) glycerol 50 mM DTT, 0.01% (m/v) bromphenol blue, 0.5% (v/v) protease inhibitor cocktail and applied for Western blotting. Protein samples were separated by 10% SDS-PAGE and transferred to a 0.45 μM pore size nitrocellulose membrane at 100 V for 1.5 h. The membranes were blocked with 5% (m/v) nonfat dry-milk powder solution in phosphate buffered saline consisting of 137 mM NaCl, 2.7 mM KCl, 10 mM Na_2_HPO_4_ and 1.8 mM KH_2_PO_4_ (PBS) plus 0.1% (v/v) Tween20 (PBST), or alternatively in Tris-buffered saline consisting of 50 mM Tris–HCl (pH 7.4), 150 mM NaCl (TBS) plus 0.1% (v/v) Tween 20 (TBST) when phospho-specific antibodies were used. During Western blotting the membranes were incubated for 3 hours at room temperature with the following antibodies at the indicated dilutions:mouse monoclonal anti-eNOS (1:2000), rabbit polyclonal anti-MYPT1 (1:1000), rabbit polyclonal-anti-GST (1:1000), rabbit polyclonal anti-MYPT1^pT696^ (1:1000), rabbit polyclonal anti-eNOS^pT497^ (1:1000), rabbit polyclonal anti-eNOS^pS1179^ (1:1000), rabbit polyclonal anti-MLC20^ppT18/S19^, mouse monoclonal anti-β-tubulin (1:2000), rabbit polyclonal anti-PP1cδ (1:1000), rabbit polyclonal anti-PKAcα (1:1000), mouse monoclonal anti-67LR (1:1000), rabbit polyclonal anti-eNOS^pY657^/nNOS^pY895^ antibody (1:1000), mouse monoclonal anti-glutathion antibody (1:1000) and rabbit polyclonal anti-PP2A-B56δ (1:1000). After incubation with the primary antibodies the membranes were washed three times with PBST or TBST and incubated with horseradish-peroxidase (HRP) conjugated goat polyclonal anti-rabbit- or rabbit polyclonal anti-mouse antibody (1:4000). Immunoreactive proteins were developed with enhanced chemiluminescence (ECL) based detection system. The signals were detected either with FluorChem AIC system (Alpha Innotec, San Jose, CA, USA) or on autoradiography films. Representative images of the Western blots were cropped from the whole blot images by Adobe Photosop CS5 software (Adobe Systems Inc., San Jose, CA, USA). Uncropped images of the Western blots are shown in Supplementary Information (Figures S1 and S8). For densitometric analysis ImageJ software (Research Services Branch, National Institute of Health (Bethesda, MD, USA) was used.

### Transfection and gene silencing

In order to overexpress MYPT1 and/or eNOS, tsA201 cells were transfected with pcDNA3.1c-myc-eNOS alone, pM11-FLAG-MYPT1 alone or co-transfected with both pcDNA3.1-myc-eNOS and pM11-FLAG-MYPT1 expression vectors, using jetPEI transfection reagent according to the description of the manufacturer. Human embryonic kidney derived tsA201 cells were plated in 6-well plates and at 70–80% confluence were incubated with DNA/jetPEI complex. In 100 μl serum free medium, 3 μg DNA and 12 μl jetPEI was mixed then the diluted transfection reagent was added to the DNA. The mixture was incubated at room temperature for 30 min and added to the cells on 6 well plates with 1.8 ml complete medium. After 48 h incubation the medium was collected for nitrite/NO measurement and the cells were lysed in 100 μl lysis buffer (1% (v/v) Triton X-100, 150 mM NaCl, 50 mM Tris–HCl (pH 7.4), 0.1% (m/v) SDS, 1% (m/v) Na-deoxycholate, 20 mM EDTA and 0.5% (v/v) protease inhibitor mix), then boiled with SDS sample buffer and applied for immunoblot analysis. In parallel experiments the media was replaced with fresh media for treatment with agonist and then collected for nitrite measurement. Overexpression of eNOS and MYPT1 was assessed in whole cell lysate by immunoblot analysis. BPAE cells seeded on 6 well plates were transfected with non-specific (scrambled) or pan PP1c, or MYPT1 specific siRNA duplexes in 20 nM final concentration using DharmaFect 2 transfection reagent. Transfection reagent and different siRNAs were diluted is serum free media in separate tubes. After 5 minutes of incubation at room temperature diluted transfection reagent was added to the diluted siRNA, followed by incubation at room temperature for 20 minutes. When the transfection complex was formed, the transfection mixture was added to BPAECs in serum free media. After 6 hours incubation FBS was supplemented to the cell cultures in 20% of final concentration, followed by 48 h further incubation. The transfected cells were washed once with ice-cold PBS, lysed in 100 μl lysis buffer and used for Western blotting.

### Nitric-oxide measurement

NO-content of the cell culture medium was determined by Sievers NO Analyzer NOA 280i (Sievers Instruments Inc., Boulder, CO) according to the manufacturer’s instructions. After 30 minutes or 48 hours of incubation the NO formed in the cells diffused to the medium and was converted to nitrite in the presence of oxygen. Protein content of cell culture medium was precipitated with 200 mM ZnSO_4._ One hundred microliters of each samples were applied to the reaction chamber, where nitrite was converted to NO by sodium iodide and was liberated by purging. The NO liberated was converted by ozone to NO_2_ and chemiluminescence of NO_2_ equivalent to NO formation was determined.

### Nitric-oxide measurement with DAF-2 DA

The BPAE cells were seeded on 13 mm diameter coverslips which were placed in 24 well plates before seeding. The cells were then cultured until 60–80% of confluence. The culturing media was then replaced with the reaction buffer, which includes 1 μM β-NADH, arginine substrate, 25 μM 4,5-diaminofluorescein diacetate (DAF-2 DA) provided by the manufacturer and the cells were incubated for 2 hours at 37 °C. Then PMA or EGCG was added to the corresponding wells for 1 hour. In parallel, non-treated coverslips (control) were also incubated with reaction buffer. Afterward the cells were washed with PBS and fixed in 4% (v/v) paraformaldehyde (PFA) solution for 10 minutes at room temperature. After washing three times with PBS the samples were mounted with 4.3 (m/v) Mowiol and prepared for confocal microscopy. The DAF-2 was excited at 488 nm, and the detection range was 500–580 nm (Munhoz *et al*.)^54^. The intensity of single cell fluorescence from 8-bit tiff images was determined using Image-J (NIH). The dye formation was calculated using the equation:


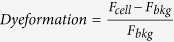
, which was based on the dye uptake equation described previously (Nagy *et al*.)^55^.

### Pull-down assays

GST-MYPT1 pull-down experiments were carried out by coupling of GST (in control experiments) or GST-MYPT1 to glutathione-Sepharose beads (Lontay *et al*.)^49^, then the HUVEC lysate was applied to the resin and incubated for 3 hours at 4 °C. The resin was washed three times with washing buffer (1 M NaCl, 20 mM Tris-HCl, pH 7.4, 0.1% (v/v) Triton X-100), then boiled for 10 minutes with SDS sample buffer and subjected to immunoblot analysis with anti-eNOS antibody. Pull-down of overexpressed c-myc-eNOS or FLAG-MYPT1 from tsA201 cell lysate were carried out using anti-c-myc and anti-FLAG M2 antibody-coupled EZview^TM^ Red affinity gel and analyzed with anti-MYPT1 and anti-eNOS or with anti-c-myc and anti-FLAG antibodies.

### *In vitro* phosphorylation/dephosphorylation assays

C-myc-eNOS was overexpressed in tsA201 cells and the cells were lysed in lysis buffer containing 1% (v/v) Triton X-100, 150 mM NaCl, 50 mM Tris–HCl (pH 7.4), 0.1% (m/v) SDS, 1% (m/v) Na-deoxycholtate, 20 mM EDTA and 0.5% (v/v) protease inhibitor mix, centrifuged (10 000 *g*, 10 min) and the supernatant was bound to EZview^TM^ Red anti-c-Myc affinity gel. The gel was washed three times with a solution containing 1% (v/v) Triton X-100, 300 mM NaCl, 50 mM Tris–HCl (pH 7.4), 0.1% (m/v) SDS, 1% (m/v) Na-deoxycholtate, 20 mM EDTA and 0.5% (v/v) protease inhibitor mix. Next, the affinity gel matrix was resuspended in ROCK assay buffer (30 mM Tris–HCl (pH 7.5), 85 mM KCl, 5 mM MgCl_2_, 1 mM EGTA, 1 mM dithiothreitol (DTT) and 1 μM microcystin-LR (MC-LR). Twenty percent of the gel was removed and boiled in hot SDS sample buffer (ROCK, 0 min). Phosphorylation of c-myc-eNOS was started by addition of 0.2 mM ATP in the presence of 5 mU/mL ROCK and the mixture was incubated for 60 min, followed by 6 washing steps with 20 mM Tris-HCl (pH 7.5) to remove MC-LR, ATP and Mg^2+^. The gel was resuspended again in 100 μl 20 mM Tris-HCl (pH 7.5) buffer and another 20% of the initial gel amount was removed and boiled in SDS sample buffer (ROCK 60 min). The ROCK-phosphorylated sample was divided into three equal aliqouts and was incubated for 15 min with 15 nM purified FLAG-MYPT1, 5 nM purified native rabbit skeletal muscle PP1c^52^ or 5 nM PP1c plus 15 nM FLAG-MYPT1. The reactions were stopped by adding hot SDS sample buffer, then the samples were boiled for 5 minutes and subjected to Western blotting.

### Assay of protein phosphatase activity

The assay of phosphatase activity of BPAECs was carried out as previously described for THP-1 cells^31^. Prior to treatments, BPAECs were incubated in serum-free medium for 16 h, then cells were untreated (control), or treated with 10 nM CLA for 30 min, or with 20 μM EGCG for 1 hour. Cells were collected by centrifugation (600 *g*, 5 min) and washed with phosphate buffered saline (PBS) followed by washing with TBS containing 0.1 mM EDTA. Cells were resuspended in 100 μl ice-cold TBS containing 0.1 mM EDTA supplemented with 0.5% (v/v) protease inhibitor cocktail and 50 mM 2-mercaptoethanol. Cells were lysed by sonication on ice, then clarified by centrifugation (13 000 g, 10 min). The phosphatase activity of the supernatants was determined at 30 °C in 20 mM Tris–HCl (pH 7.4) and 0.1% (v/v) 2-mercaptoethanol with 1 μM ^32^P-MLC20 in the absence or presence of 2 μM His-inhibitor-2 (I2)^31^. The reaction was initiated by addition of the substrate. After 10-min incubation, the reaction was terminated by the addition of 200 μl 10% TCA and 200 μl 6 mg/ml BSA. The precipitated proteins were collected by centrifugation and the released ^32^P_i_ was determined from the supernatant (370 μl) in a scintillation counter.

### Immunofluorescence and confocal microscopy

BPAECs and HPAECs were plated on gelatin-coated glass coverslips and were grown for 24 hours. After fixation with 4% (v/v) PFA for 10 minutes, the cells were permeabilized with 0.1% (v/v) Triton X-100, 4% (m/v) BSA and 0.01% (m/v) NaN_3_ in PBS (pH 7.5) for 1 hour followed by three washing steps with 4% (m/v) BSA in PBS to block the nonspecific binding sites and once with antibody diluting solution (0.1% (v/v) Triton X-100, 0.1% (m/v) BSA and 0.01% (m/v) NaN_3_ in PBS (pH 7.5)). After blocking the cells were incubated overnight at 4 °C with mouse monoclonal anti-eNOS in 1:250, or with rabbit polyclonal anti-MYPT1^1–296^ antibody in 1:100 dilution. Next, the cells were washed gently (three times) with PBS and incubated with goat polyclonal Alexa-488 (1:250) or goat polyclonal Alexa-546 (1:250) conjugated secondary antibodies for 1 hour at room temperature. Finally, the cells were washed three times with PBS and covered in Prolong Gold Antifade mounting medium. The localization of the specifically labelled eNOS and MYPT1 was visualized using a confocal microscope (Olympus Fluoview 1000, Hamburg, Germany) equipped with 60X UPLSAPO (NA 1.35) oil immersion objective, or with Leica X8 confocal microscope (Leica Microsystems CMS GmbH, Mannheim, Germany). The optical thickness of the co-localization images was 1 μm.

### Duolink poximity ligation assay

BPAECs cultured in 24 well plates on glass coverslips were fixed with 4% PFA solution for 10 minutes, permeabilized with 0.1% (v/v) Triton X-100 for 10 minutes and blocked with 5% (m/v) BSA in PBS. The proximity ligation assay (PLA) was carried out according to the manufacturer’s instructions. Briefly, to visualize the interaction between eNOS and MYPT1 the samples were stained with mouse anti-eNOS (1:400) and rabbit anti-MYPT1^1–296^ (1:500) antibodies overnight, at 4 °C prior to incubation with PLA probes for 1 hour at 37 °C. Then, ligase was added to hybridize the probes and after the rolling-circle amplification (RCA) the labelled probes were hybridized to the RCA product. After washing three times with PBS the samples were mounted with 4.3 (m/v) Mowiol and prepared for confocal microscopy.

### Surface plasmon resonance measurement

Interaction of GST-MYPT1 with c-myc-eNOS was analyzed by surface plasmon resonance based binding experiments using a Biacore 3000 instrument (GE Healthcare, Uppsala, Sweden) as described previously (Kolozsvári *et al*.)^25^. Anti-GST was immobilized on CM5 sensor chip by amine-coupling according to the instructions of the manufacturers. On one surface recombinant GST while on the two other surfaces full-length GST-MYPT1 were immobilized in running buffer containing 10 mM Hepes (pH 7.4), 0.15 M NaCl, 3 mM EDTA, and 0.005% (v/v) Surfactant P20. C-myc-eNOS at 0.5 μM or 1 μM in running buffer was injected over the surfaces and the amount of the captured analyte was determined from the changes of the resonance signal expressed as response units (RU). The surface (with immobilized recombinant GST) was treated identically to the GST-MYPT1 surfaces to determine unspecific binding which was subtracted from the data obtained with the GST-MYPT1 surfaces.

### Transendothelial permeability measurement

Transendothelial electrical resistance (TER) of BPAECs was measured using electric cell-substrate impedance sensing (ECIS) instrument, Model 1600 R (Applied BioPhysics, Troy, NY, USA) as described (Bogatcheva *et al*.)^56^. Approximately equal numbers (3.5 × 10^4^/well) of BPAE cells were seeded on electrode arrays (8W10E) and the experiments were performed on wells that achieved > 1000 Ω of baseline steady-state resistance. One hour before treating the cells the media was changed to serum-free MEM and the changes in the initial resistance were measured, then after the different treatments with PMA, CLA TM or EGCG the data were normalized to the initial resistance values and plotted as normalized resistance.

### Statistical analysis

Statistical significance of differences measured between control and treated samples were determined by Student’s *t*-test for two groups or by analysis of variance (ANOVA) followed by Newman-Keuls post hoc testing. Results with *P* < 0.05 were considered statistically significant.

## Additional Information

**How to cite this article:** Bátori, R. *et al*. Interplay of myosin phosphatase and protein phosphatase-2A in the regulation of endothelial nitric-oxide synthase phosphorylation and nitric oxide production. *Sci. Rep.* **7**, 44698; doi: 10.1038/srep44698 (2017).

**Publisher's note:** Springer Nature remains neutral with regard to jurisdictional claims in published maps and institutional affiliations.

## Supplementary Material

Supplementary Information
