# Supplementary material for: Interplay of myosin phosphatase and protein phosphatase-2A in the regulation of endothelial nitric-oxide synthase phosphorylation and nitric oxide production
Source: Sci Rep. 2017 Mar 16;7:44698. doi: 10.1038/srep44698 (PMC5353758; doi:10.1038/srep44698)
Supplement: Supplementary Information [file srep44698-s1.pdf]

Supplementary Information

Interplay of myosin phosphatase and protein phosphatase-2A in the regulation of endothelial nitric-oxide synthase phosphorylation and nitric oxide production

Róbert Bátori[1], Bálint Bécsi[1, 2], Dénes Nagy[1], Zoltán Kónya[1], Csaba Hegedűs[1], Zsuzsanna Bordán[3], Alexander Verin[3], Beáta Lontay[1], Ferenc Erdődi[1, 2 *]

[1]Department of Medical Chemistry and [2]MTA-DE Cell Biology and Signaling Research Group, Faculty of Medicine, University of Debrecen, Debrecen, Egyetem tér 1, H-4032 Hungary; [3]Vascular Biology Center, Division of Pulmonary Medicine, Medical College of Georgia, Augusta University, Augusta, GA, USA.

***Correspondence to:**

Dr. Ferenc Erdődi

Department of Medical Chemistry,

Faculty of Medicine, University of Debrecen

Debrecen, Egyetem tér 1.

H-4032 Hungary

Tel.: +36 52 412345; fax: +36 52 412566;

e-mail: erdodi@med.unideb.hu

Materials and Methods

Materials

Chemicals and vendors were as follows. Fetal bovine serum, antibiotic-antimycotic solution, sodium pyruvate, L-glutamine, Minimum Essential Medium (MEM) and Dulbecco's modified Eagle's medium (DMEM) (Gibco Gaithersburg, MD, USA). M199 media, HEPES buffered saline, amphotericin B, penicillin-streptomycin and trypsin-EDTA solution, anti-FLAG M2 antibody-coupled EZview™ Red affinity gel, anti-FLAG antibodies, EZview™ Red anti-c-Myc affinity gel, anti-c-myc antibody, horseradish-peroxidase (HRP) conjugated anti-mouse IgG and HRP-conjugated anti-rabbit IgG, mycrocistin-LR, phorbol 12-myristate-13-acetate (PMA), Duolink® in situ red kit, Mowiol and Fluorimetric nitric oxide synthase detection kit (Sigma-Aldrich, St Louis, MO, USA). Endothelial basal medium-2 (EBM-2) and EBM-2 SingleQuot Kit (Clonetics San Diego, CA, USA). Complete mini protease inhibitor Cocktail Tablets (Roche Diagnostics, Mannheim, Germany). jetPEI transfection reagent (Polyplus Transfections, Illkirche, France). Precision Plus Protein™ Dual Color Standard and 0.45 μM pore size nitrocellulose membrane (Bio-Rad, Hercules, CA, USA). Enhanced chemiluminescence (ECL) reagent (Pierce, Rockford, IL). Protein-A Sepharose and glutathione-Sepharose beads (Amershem Bioscience, Arlington Heights, IL). Anti-MYPT1^{pT696}, anti-PP1c, anti-eNOS^{pT497}, anti-eNOS^{pS1179}, anti-PKAcα, anti-MLC20$^{ppT18/S19}$ and anti-GST antibody (Cell Signaling, Beverly, MA, USA). Anti-67LR, anti-PP2A-B56δ and anti-glutathion antibody (Abcam, Cambridge, MA, USA). Anti-eNOS^{pY657}/nNOS^{pY895} antibody (ECM Bioscience, Versailles, KY). ProLong Gold Antifade mounting medium, Alexa-488 anti-rabbit IgG and Alexa-546 anti-mouse IgG (Molecular Probes, Eugene, OR, USA). Non-specific (scrambled) and pan PP1c specific siRNA (Santa Cruz Biotechology Inc., Santa Cruz, CA). MYPT1 and siPKAcα specific siRNA duplexes (Dharmacon Research, Lafayette, CO, USA). DharmaFect 2 transfection reagent (Thermo Scientific, Waltham, MA

USA). Tautomycin (TM) and anti-β-tubulin antibody (Millipore, Solna, Sweden). Calyculin A (CLA) (Calbiochem, Darmstadt, Germany). pcDNA3.1c-myc-eNOS (kind gift of Prof. Dr. Stefanie Dimmeler, University of Frankfurt); pM11-FLAG-MYPT1 (GeneCopoeia, Rockville, MD, USA). Anti-eNOS antibody (BD Transductional Laboratories, San Jose, CA, USA). Anti-PP1cδ and ROCK (Upstate, Lake Placid, NY). CM5 sensor chips and anti-GST capture kit (GE Healthcare, Uppsala, Sweden). HyBlot® ES autoradiography film (Denville Scientific Inc., Holliston, MA, USA).

Supplementary Figures and Legends

S1

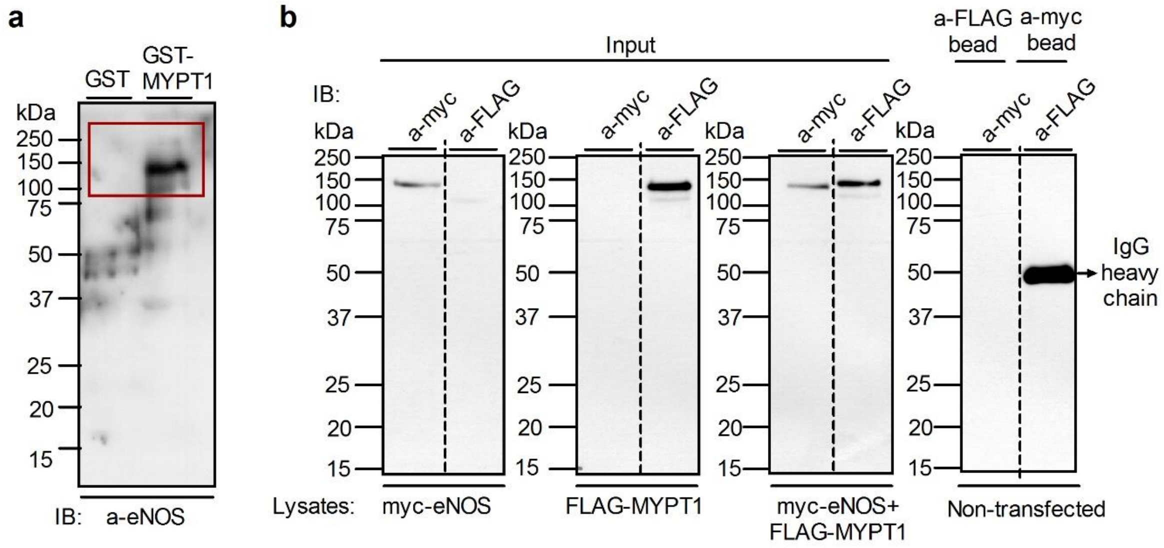

Figure S1: (**a**) Image of the uncropped Western blot of GST-MYPT1 pull-down assay. (**b**) Representative Western blots demonstrating overexpression of c-myc-eNOS and FLAG-MYPT in tsA201 cells. Cells were transfected with constructs for c-myc-eNOS alone, FLAG-MYPT1 alone or with c-myc-eNOS and FLAG-MYPT1 together and developed with anti-c-myc (a-c-myc) and anti-FLAG (a-FLAG) antibodies (left panel). Nontransfected tsA201 cell lysates were subjected to pull-down experiments using anti-FLAG and anti-c-myc beads and the samples were analysed with anti-myc and anti-FLAG antibodies (right panel).

S2

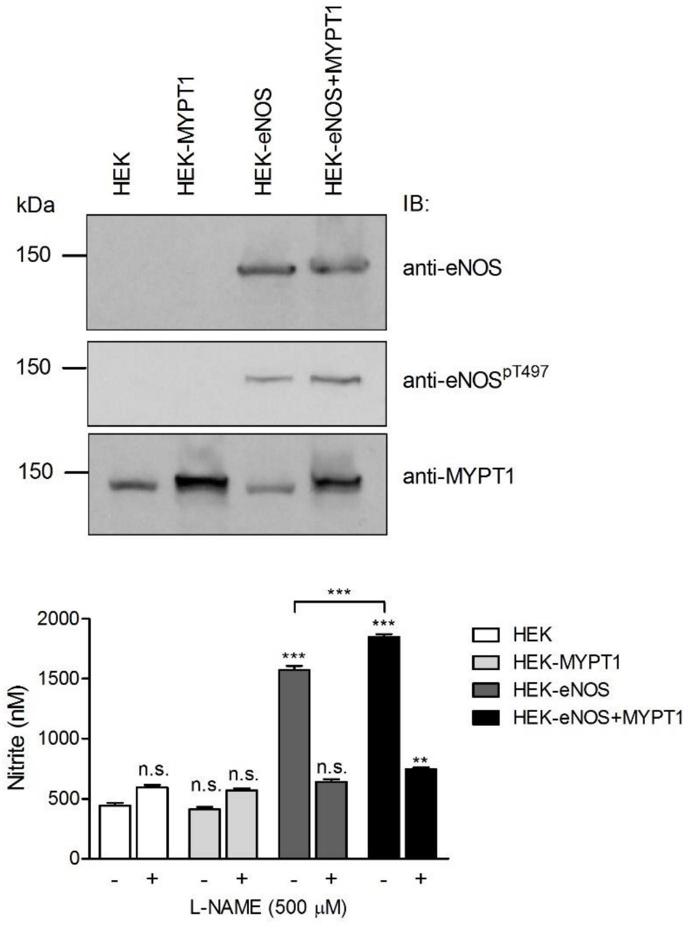

Figure S2: N-nitro-L-arginine-methyl ester (L-NAME) inhibits eNOS activity and NO production in HEK-eNOS knock-in cells in the absence or in the presence of FLAG-MYPT1 overexpression. **Upper panel**: identification of eNOS, eNOS^{pThr497} and MYPT1 by Western blotting in non-transfected (HEK) and FLAG-MYPT1 transfected (HEK-MYPT1), or in HEK293-eNOS knock-in cells without (HEK-eNOS) or with FLAG-MYPT1 overexpression (HEK-eNOS+MYPT1). **Lower panel**: Approximately equal number of HEK, HEK-MYPT1, HEK-eNOS and HEK-eNOS+MYPPT1 cells were plated in 24 well plates and incubated in the absence or the presence of 500 µM L-NAME for 24 hours, then NO was determined in the culture medium. Data represent means ± SEM (n=3), n.s.: not significant, $^{**}p < 0.01$, $^{***}p < 0.001$, One-way ANOVA, Newman-Keuls post-hoc testing.

S3

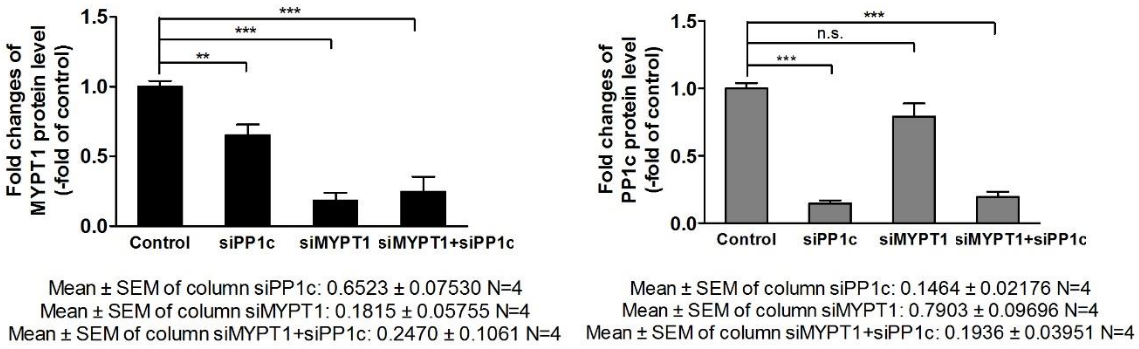

Figure S3: Statistical analysis of MYPT1 and PP1cδ silencing efficiency in BPAECs. Bar graphs represent changes in the protein level of MYPT1 (left panel) and PP1cδ (right panel) determined by densitometric analysis of blots from at least four independent experiments (means ± SEM, n.s.: not significant, **p*<0.05, ***p*<0.01, *** *p*<0.001 compared to control, One-way ANOVA, Newman-Keuls post-hoc testing).

S4

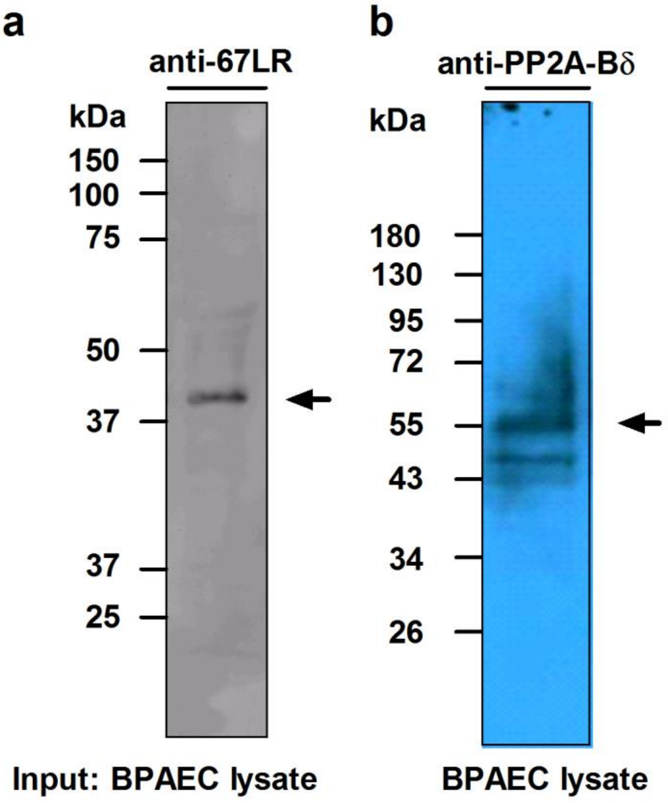

Figure S4: Identification of 67-kDa laminin receptor and the B56δ subunit of PP2A (PP2A-Bδ) in BPAE cells by Western blotting. The antibodies gave cross-reactions according to the same pattern described by the suppliers.

S5

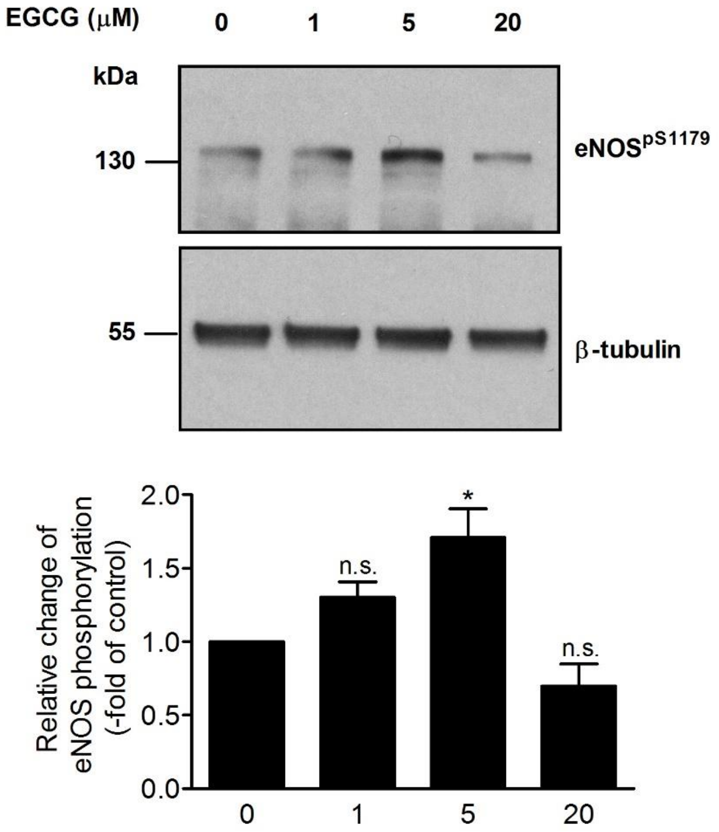

Figure S5: The effect of EGCG treatment on the level of $eNOS^{pSer1179}$. BPAECs were treated as described in Fig. 6 (**a**) and changes in the level of $eNOS^{pSer1179}$ was assessed by Western blotting with phospho-specific antibody (anti- $eNOS^{pSer1179}$) upon the challenges at different concentration of EGCG (upper panel). Bar graphs (lower panel) represent the changes in the level $eNOS^{pSer1179}$ determined by densitometric analysis of blots from 3 independent experiments (means ± SEM, n.s.: not significant, *p<0.05 compared to control, i.e. without EGCG, One-way ANOVA, Newman-Keuls post-hoc testing).

S6

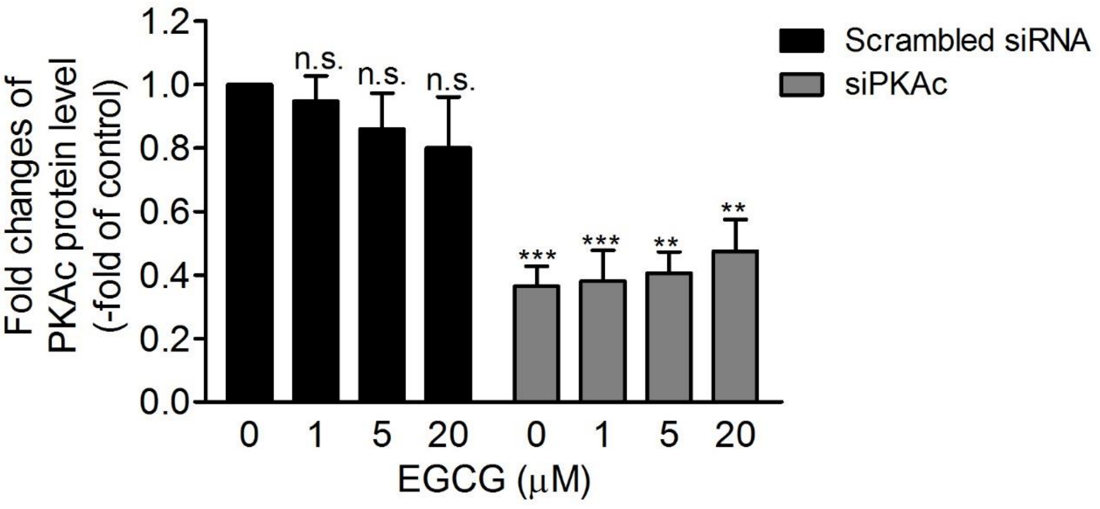

Mean ± SEM of column 0 μM EGCG siPKAc: 0.3646 ± 0.06275 N=4
Mean ± SEM of column 1 μM EGCG siPKAc: 0.3807 ± 0.09708 N=4
Mean ± SEM of column 5 μM EGCG siPKAc: 0.4051 ± 0.06747 N=4
Mean ± SEM of column 20 μM EGCG siPKAc: 0.4745 ± 0.09990 N=4

Figure S6: Statistical analysis of PKAcα silencing efficiency in BPAECs. Bar graphs represent changes in the protein level of PKAcα determined by densitometric analysis of blots from four independent experiments (means ± SEM, n.s.: not significant, ***p*<0.01, *** *p*<0.001 compared to scrambled siRNA-transfected without EGCG treatment as control, One-way ANOVA, Newman-Keuls post-hoc testing).

S7

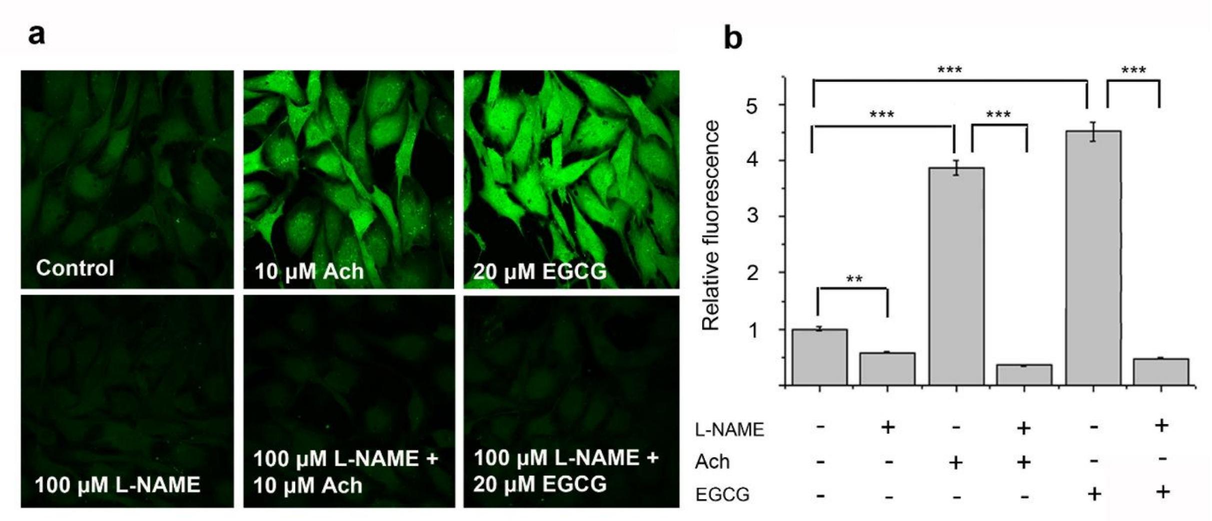

Figure S7: Effect of N-nitro-L-arginine-methyl ester (L-NAME) on the acetylcholin (Ach) or EGCG induced increase in NO in BPAECs. (**a)** Representative images of NO detection with DAF-2 fluorescent dye in BPAECs after distinct treatments indicated on the images. BPAECs were preincubated without (upper images) or with (lower images) 100 µM L-NAME for 60 min then challenged with 10 µM Ach or 20 µM EGCG for 60 min. (**b)** Single cell fluorescence determined by ImageJ after different treatments as indicated. Results of single cell values (80 to 90 individual cells were analysed for each treatment) of two independent experiments are shown as means ± SEM. (**p<0.01; *** p<0.001, One-way ANOVA, Holm-Slidak post-hoc testing).

S8

Figure 2a

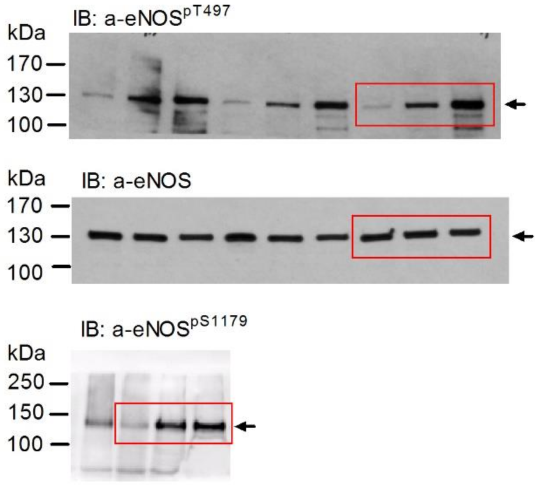

Figure 2b

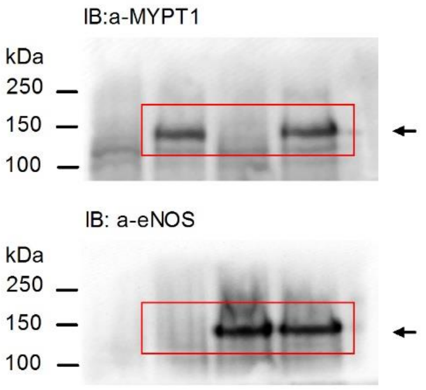

Figure 3

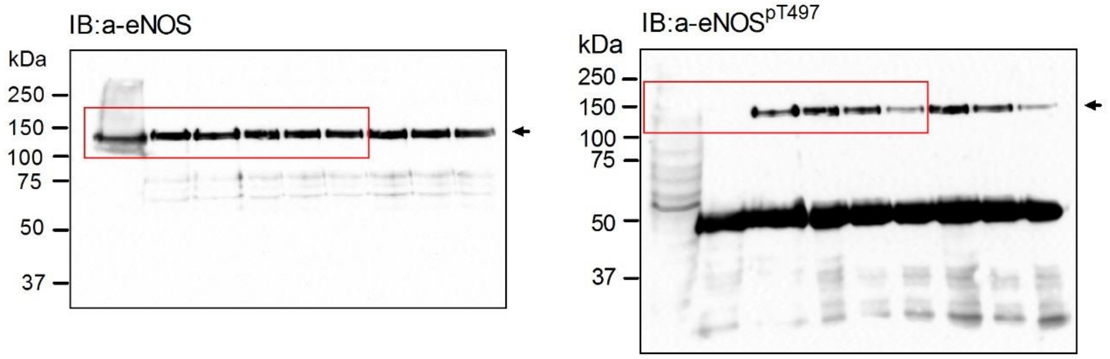

Figure 4

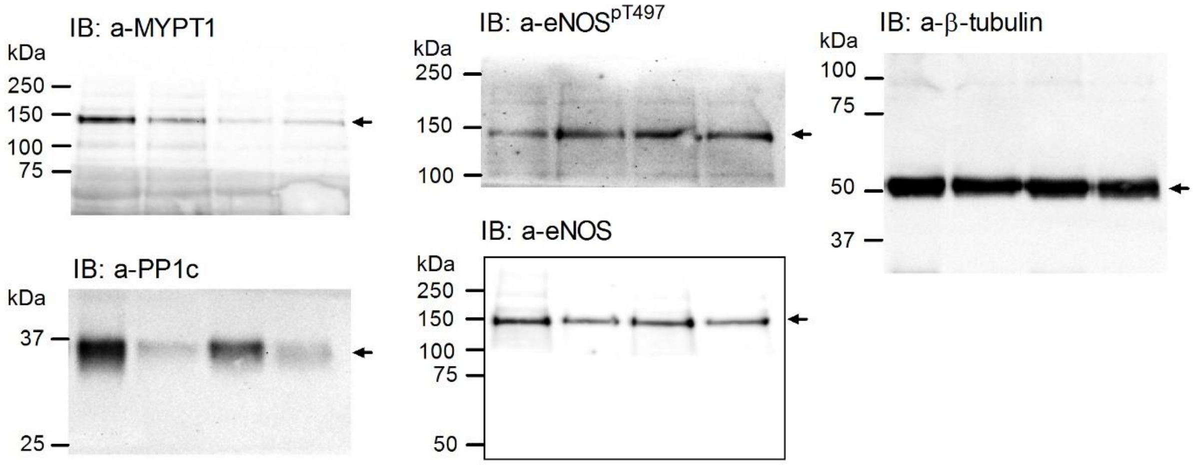

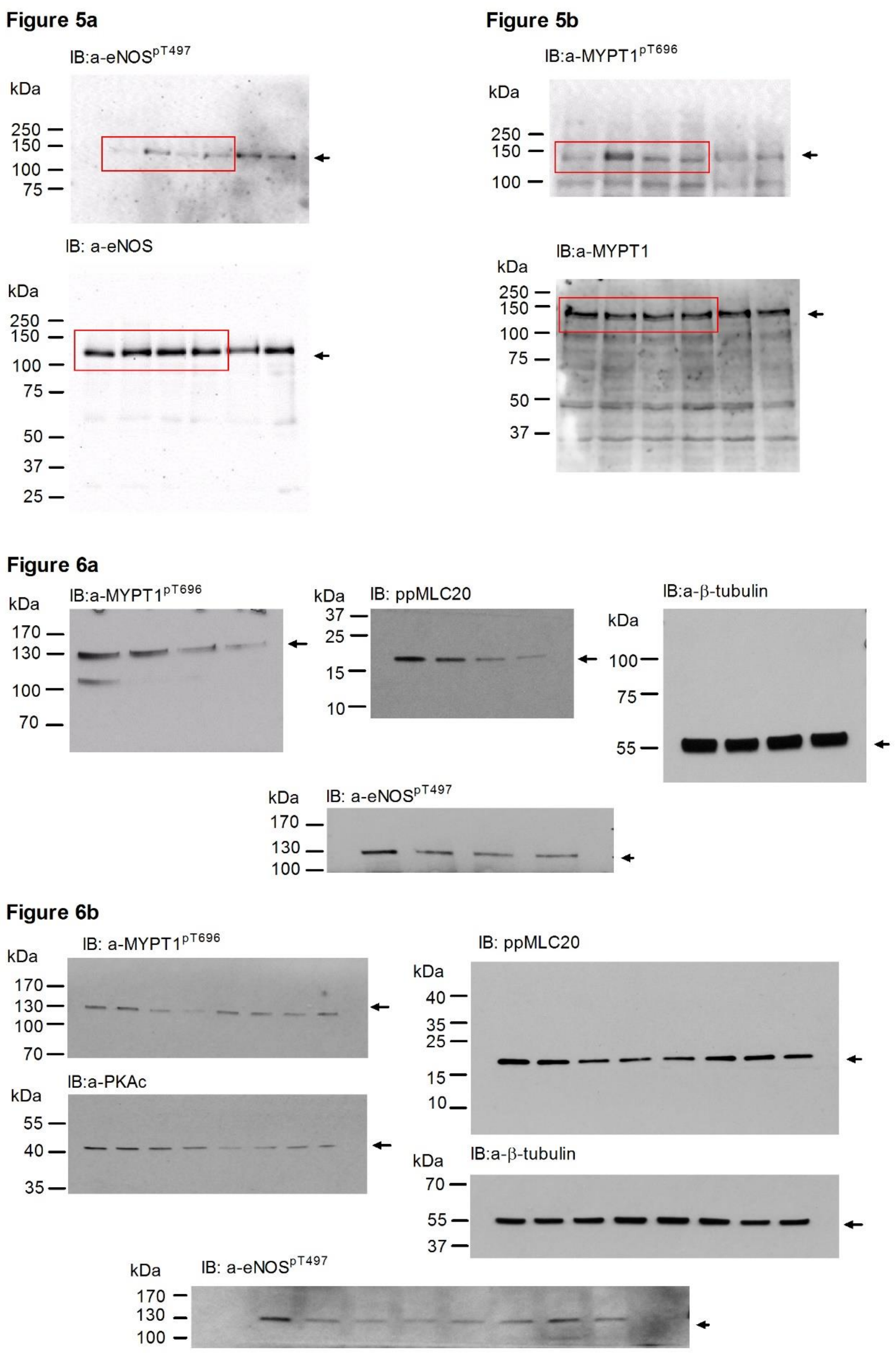

Figure S8: Full-length blots of the representative cropped images in Figs. 2-6. Note that in cases where only parts of the blots are presented the membranes were cut into pieces in order to identify proteins of distinct molecular mass on the same blots.
